# Avian influenza overview June–September 2023

**DOI:** 10.2903/j.efsa.2023.8328

**Published:** 2023-10-05

**Authors:** European Food Safety Authority, European Centre for Disease Prevention and Control, European Union Reference Laboratory for Avian Influenza, Cornelia Adlhoch, Alice Fusaro, José L Gonzales, Thijs Kuiken, Gražina Mirinavičiūtė, Éric Niqueux, Christoph Staubach, Calogero Terregino, Francesca Baldinelli, Alessia Rusinà, Lisa Kohnle

**Keywords:** avian influenza, captive birds, HPAI, humans, monitoring, poultry, wild birds

## Abstract

Between 24 June and 1 September 2023, highly pathogenic avian influenza (HPAI) A(H5) outbreaks were reported in domestic (25) and wild (482) birds across 21 countries in Europe. Most of these outbreaks appeared to be clustered along coastlines with only few HPAI virus detections inland. In poultry, all HPAI outbreaks were primary and sporadic with most of them occurring in the United Kingdom. In wild birds, colony‐breeding seabirds continued to be most heavily affected, but an increasing number of HPAI virus detections in waterfowl is expected in the coming weeks. The current epidemic in wild birds has already surpassed the one of the previous epidemiological year in terms of total number of HPAI virus detections. As regards mammals, A(H5N1) virus was identified in 26 fur animal farms in Finland. Affected species included American mink, red and Arctic fox, and common raccoon dog. The most likely source of introduction was contact with gulls. Wild mammals continued to be affected worldwide, mostly red foxes and different seal species. Since the last report and as of 28 September 2023, two A(H5N1) clade 2.3.4.4b virus detections in humans have been reported by the United Kingdom, and three human infections with A(H5N6) and two with A(H9N2) were reported from China, respectively. No human infection related to the avian influenza detections in animals on fur farms in Finland or in cats in Poland have been reported, and human infections with avian influenza remain a rare event. The risk of infection with currently circulating avian H5 influenza viruses of clade 2.3.4.4b in Europe remains low for the general population in the EU/EEA. The risk of infection remains low to moderate for occupationally or otherwise exposed people to infected birds or mammals (wild or domesticated); this assessment covers different situations that depend on the level of exposure.

**Requestor:** European Commission

**Question number:** EFSA-Q-2023-00091 and Commission request 280 to ECDC (SANTE.B.2/IK/mo (2023)2182203)

**Acknowledgements:** In addition to the listed authors, EFSA, ECDC and the EURL wish to thank the Member State representatives who provided epidemiological data on avian influenza outbreaks or shared sequence data: Sandra Revilla-Fernández, Janna Vogelgesang and Irene Zimpernik (Austria); Ingeborg Mertens, Mieke Steensels, Steven Van Borm and Philippe Houdart (Belgium); Aleksandra Miteva (Bulgaria); Tihana Miškić and Savić Vladimir (Croatia); Vasiliki Christodoulou and Georgios Krasias (Cyprus); Alexander Nagy and Luděk Závada (Czechia); Charlotte Kristiane Hjulsager and Michelle Fischer Carlsen (Denmark); Kärt Jaarma and Imbi Nurmoja (Estonia); Tuija Gadd, Ari Kauppinen, Lauri Kareinen, Niina Tammiranta, Tiia Tuupanen, Sirpa Kiviruusu and Hanna Lounela (Finland); Camille Boyer, Karen Bucher, Béatrice Grasland, Célia Locquet, Audrey Schmitz and François-Xavier Briand (France); Anne Pohlmann, Timm Harder and Carola Sauter-Louis (Germany); Sokratis Perdikaris (Greece); Brigitte Brugger (Iceland); Laura Garza Cuartero and Audrey Jenkinson (Ireland); Paolo Mulatti, Francesca Scolamacchia, Isabella Monne, Bianca Zecchin, Ambra Pastori, Diletta Fornasiero and Luca Martelli (Italy); Māra Užule (Latvia); Vilija Grigaliūnienė and Marius Masiulis (Lithuania); Vicky Bink and Chantal Snoeck (Luxembourg); Marcel Spierenburg, Nancy Beerens, Arnout de Bruin and Dennis Bol (Netherlands); Siri Margrete Løtvedt and Britt Gjerset (Norway); Aleksandra Podrażka, Krzysztof Śmietanka, Katarzyna Domańska-Blicharz and Edyta Swieton (Poland); Renata Carvalho (Portugal); Flavius Prelipcean and Iuliana Onita (Romania); Martin Chudy, Barbora Pavlíková, Martin Tinak and Zuzana Dirbakova (Slovakia); Aleksandra Hari and Brigita Slavek (Slovenia); Monserrat Agüero García, Germán Cáceres Garrido, Luis José Romero Gonzalez, Elena García Villacieros and Azucena Sánchez (Spain); Malin Grant and Siamak Zohari (Sweden); Claudia Bachofen, Dominique Suter and Alexandra Briner (Switzerland); Susan Cusack (United Kingdom (Northern Ireland)). The authors also wish to thank Ron Fouchier from Erasmus Medical Center (Netherlands); Ian Brown from the Animal and Plant Health Agency (United Kingdom); Camille Delavenne from Ausvet Europe for conducting the data analysis under the contract OC/EFSA/ALPHA/2021/02; Linnea Lindgren Kero for the support provided under the contract OC/EFSA/DATA/2021/01; the authors, originating and submitting laboratories of the sequences from GISAID's EpiFlu™ Database, which is used for this assessment (Annex D); Paolo Calistri, Christian Gortázar and Karl Ståhl from EFSA's Panel on Animal Health and Welfare (AHAW); Edoardo Colzani from ECDC as well as Inma Aznar from EFSA for the support provided to this scientific output.

**Declarations of interest:** If you wish to access the declaration of interests of any expert contributing to an EFSA scientific assessment, please contact interestmanagement@efsa.europa.eu

**Suggested citation:** EFSA (European Food Safety Authority), ECDC (European Centre for Disease Prevention and Control), EURL (European Union Reference Laboratory for Avian Influenza), Adlhoch C, Fusaro A, Gonzales JL, Kuiken T, Mirinavičiūtė G, Niqueux É, Staubach C, Terregino C, Baldinelli F, Rusinà A and Kohnle L, 2023. Scientific report: Avian influenza overview June–September 2023. EFSA Journal 2023;21(10):8328, 54 pp. https://doi.org/10.2903/j.efsa.2023.8328

**ISSN:** 1831-4732

**Maps disclaimer:** The designations employed and the presentation of material on any maps included in this scientific output do not imply the expression of any opinion whatsoever on the part of the European Food Safety Authority concerning the legal status of any country, territory, city or area or of its authorities, or concerning the delimitation of its frontiers or boundaries.

The EFSA Journal is a publication of the European Food Safety Authority, an agency of the European Union.

## Table of Contents

Abstract ..... 1
1. Introduction ..... 6
2. Assessment ..... 6
2.1 HPAI virus detections in birds ..... 6
2.1.1 HPAI virus detections in birds in Europe ..... 6
2.1.2 HPAI virus detections in birds outside Europe ..... 20
2.1.3 Genetic characteristics of HPAI viruses of the A(H5N1) subtype circulating in Europe in avian species ..... 23
2.2 HPAI virus detections in non-human mammals ..... 24
2.2.1 HPAI virus detections in non-human mammals worldwide ..... 24
2.2.2 Genetic characteristics of HPAI viruses of the A(H5N1) subtype circulating in Europe in non-human mammals ..... 29
2.3 Avian influenza virus infections in humans ..... 30
2.3.1 Most recent human infections with avian influenza A(H3N8), A(H5N1), A(H5N6) and A(H9N2) viruses ..... 30
2.3.2 Human A(H3N8) cases, summary ..... 31
2.3.3 Human A(H5N1) cases, summary ..... 31
2.3.4 Human A(H5N6) cases, summary ..... 32
2.3.5 Human A(H9N2) cases, summary ..... 33
2.3.6 Genetic characteristics of HPAI viruses of the A(H5Nx) subtype from humans ..... 34
2.3.7 Additional information and international risk assessments ..... 34
2.3.8 ECDC risk assessment ..... 35
3. Conclusions ..... 37
3.1 Birds ..... 37
3.2 Mammals ..... 38
3.3 Humans ..... 39
4. Options for response ..... 39
4.1 Birds ..... 39
4.2 Mammals ..... 39
4.3 Humans ..... 40
References ..... 41
Appendix A – Terms of Reference ..... 49
A.1. Background and Terms of Reference as provided by the requestor ..... 49
A.2 Interpretation of the Terms of Reference ..... 50
Appendix B – Data and Methodologies ..... 52
B.1 Data on animals ..... 52

B.1.1 Overview of avian influenza outbreaks in Europe (ToR 1 and ToR 2) ....................................... 52
B.1.2 Overview of avian influenza outbreaks in other countries not reporting via ADIS (ToR 4) ..... 52
B.1.2 Genetic characterisation of avian influenza viruses: description of the nomenclature of the HPAI A(H5) viruses used in the document ........................................................................................................ 52
B.2 Data on humans .....................................................................................................................52

## 1. Introduction

This Scientific Report provides an overview of highly pathogenic avian influenza (HPAI) virus detections in poultry[1], captive[2] and wild birds that occurred in and outside Europe between 24 June and 1 September 2023, as well as HPAI virus detections in mammals (up to 15 September 2023) and human cases due to avian influenza virus (up to 14 September 2023).

The background, Terms of Reference and interpretation thereof are described in Appendix A, whereas the data and methodologies used are reported in Appendix B.

Scientific species names of wild birds and mammals mentioned in this report are listed in Table A.3 (Annex A) and Table 2, respectively.

## 2. Assessment

### 2.1 HPAI virus detections in birds

#### 2.1.1 HPAI virus detections in birds in Europe

Figure 1 shows all HPAI outbreaks in birds that were reported via the European Union (EU) Animal Disease Information System (ADIS) or the World Organisation for Animal Health (WOAH) World Animal Health Information System (WOAH-WAHIS) in Europe for the last six and the current epidemiological years[3] by month of suspicion. For the current epidemiological year 2022–2023, starting on 1 October 2022, data reported are truncated on 1 September 2023.

[1] According to Regulation (EU) 2016/429 of the European Parliament and of the Council of 9 March 2016, point (9), 'poultry' means birds that are reared or kept in captivity for: (a) the production of: (i) meat; (ii) eggs for consumption; (iii) other products; (b) restocking supplies of game birds; (c) the purpose of breeding of birds used for the types of production referred to in points (a) and (b).

[2] According to Regulation (EU) 2016/429 of the European Parliament and of the Council of 9 March 2016, point (10), 'captive birds' means any birds other than poultry that are kept in captivity for any reason other than those referred to in point (9), including those that are kept for shows, races, exhibitions, competitions, breeding or selling.

[3] In this document an 'epidemiological year' refers to the period starting in week 40 (the beginning of October) and ending in week 39 (the end of September) of the following year, based on the dates on which the first HPAI virus detections were observed in wild birds in Europe in 2016–2017, 2020–2021 and 2021–2022.

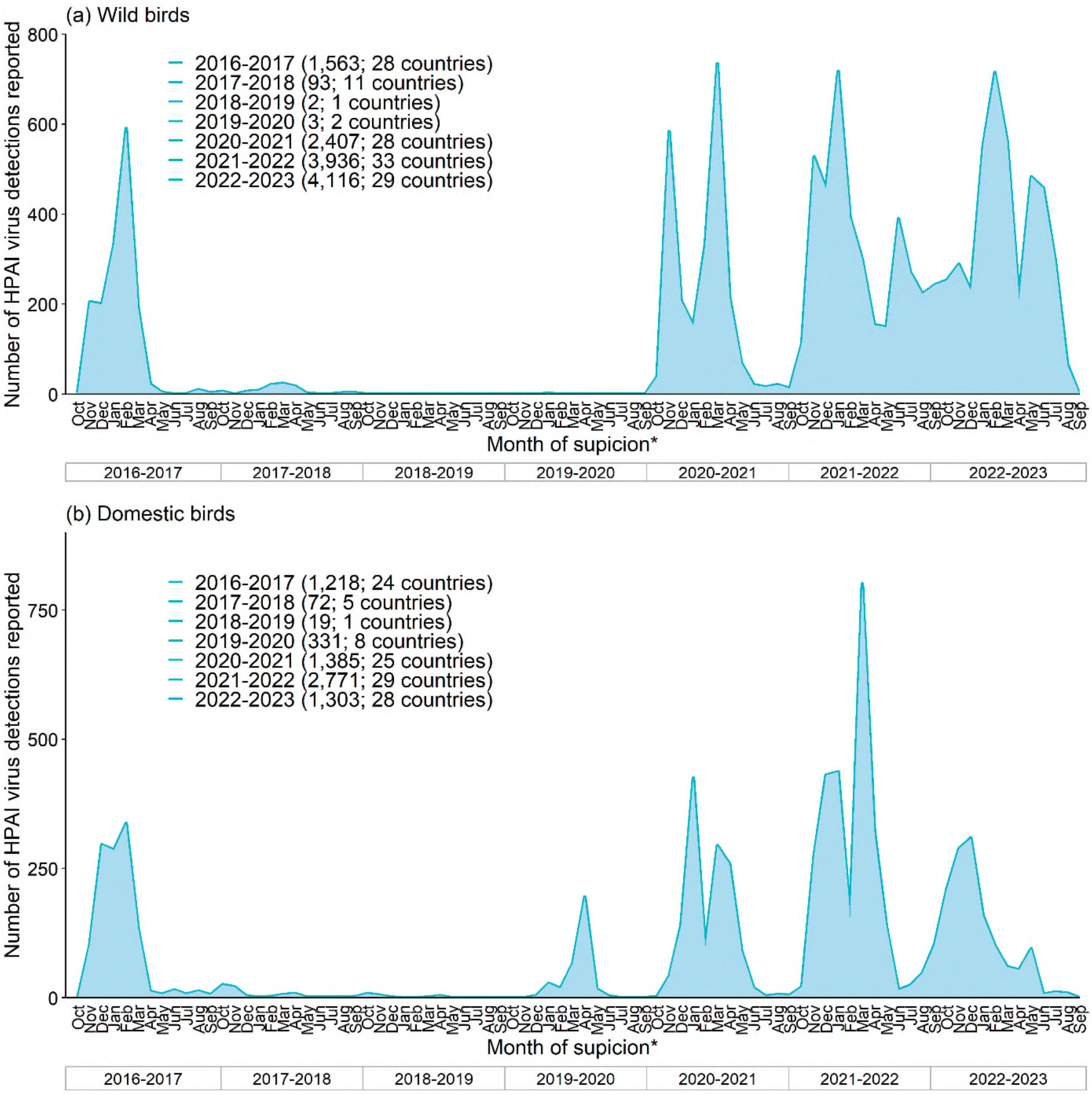

*If the date of suspicion was not available, the date of confirmation was used to assign the week of suspicion. United Kingdom data are from the Animal Disease Notification System (ADNS, former ADIS) up to 31 December 2020. From 1 January 2021 onwards, the data source was WOAH for the United Kingdom (excluding Northern Ireland) and ADNS/ADIS for the United Kingdom (Northern Ireland)[4].
Source: ADNS/ADIS and WOAH (data extraction carried out on 1 September 2023).

**Figure 1:** Distribution of the number of HPAI virus detections in wild (12,120) (a) and domestic (7,116) (b) birds reported in Europe during seven epidemiological years by month of suspicion, from 1 October 2016 to 1 September 2023 (19,236)

The HPAI epidemic observed in wild birds in the 2022–2023 epidemiological year, although still ongoing, has already surpassed the previous epidemiological year (2021–2022) in terms of total number of HPAI virus detections reported in wild birds (4,116 vs 3,936) (Figure 1a, Table 1). In addition, numbers are likely to still increase until the end of September, also due to a delay in outbreak reporting. The temporal behaviour of this epidemiological year is however similar to that observed in 2021–2022.

[4] In accordance with the Agreement on the Withdrawal of the United Kingdom from the EU, and in particular with the Protocol on IE/NI, the EU requirements on data sampling are also applicable to the United Kingdom (Northern Ireland).

**Table 1:** Number of HPAI outbreaks in Europe, by country, virus subtype and affected sub-population, from 24 June to 1 September 2023. Cumulative numbers since the start of the 2022–2023 epidemiological year are reported in parentheses (1 October 2022 to 1 September 2023)

| Reporting country | Captive birds | Poultry | | Wild birds | | | | Total |
|---|---|---|---|---|---|---|---|---|
| | A(H5N1) | A(H5Nx) | A(H5N1) | A(H5Nx) | A(H5N5) | A(H5N1) | A (Not typed) | |
| Austria | 0 (5) | - | 0 (3) | 0 (12) | - | 1 (120) | - | 1 (140) |
| Belgium | 0 (22) | - | 0 (11) | 4 (9) | - | 21 (231) | - | 25 (273) |
| Bulgaria | - | 0 (1) | 0 (2) | - | - | - | - | 0 (3) |
| Croatia | - | - | 0 (1) | - | - | 0 (5) | - | 0 (6) |
| Cyprus | 0 (1) | - | 0 (1) | - | - | 0 (1) | - | 0 (3) |
| Czechia | 0 (1) | - | 0 (32) | - | - | 1 (25) | - | 1 (58) |
| Denmark | 0 (2) | - | 1 (7) | 1 (2) | - | 17 (125) | - | 19 (136) |
| Estonia | - | - | 0 (1) | 1 (1) | - | 5 (10) | - | 6 (12) |
| Finland | - | - | - | 1 (2) | - | 23 (26) | - | 24 (28) |
| France | 1 (70) | 0 (2) | 3 (380) | 3 (3) | - | 33 (454) | - | 40 (909) |
| Germany | 0 (130) | - | 1 (67) | 0 (4) | - | 106 (1143) | - | 107 (1,344) |
| Hungary | 0 (2) | - | 0 (168) | - | - | 4 (40) | - | 4 (210) |
| Iceland | - | - | - | - | - | 0 (5) | - | 0 (5) |
| Ireland | 1 (3) | - | 0 (2) | 0 (1) | - | 8 (28) | - | 9 (34) |
| Italy | 0 (1) | - | 1 (40) | 0 (1) | - | 22 (250) | - | 23 (292) |
| Latvia | - | - | - | - | - | 13 (67) | - | 13 (67) |
| Lithuania | 0 (1) | - | - | - | - | 1 (52) | 1 (1) | 2 (54) |
| Luxembourg | 0 (1) | - | - | - | - | 0 (5) | - | 0 (6) |
| Moldova | - | - | 0 (2) | - | - | - | - | 0 (2) |
| Netherlands | 0 (15) | - | 1 (23) | - | - | 59 (463) | - | 60 (501) |
| North Macedonia | 0 (1) | - | - | - | - | - | - | 0 (1) |
| Norway | 0 (2) | - | 0 (2) | 5 (7) | 0 (3) | 23 (59) | 1 (1) | 29 (74) |
| Poland | 1 (2) | - | 1 (92) | - | - | 9 (150) | - | 11 (244) |
| Portugal | - | - | - | - | - | 0 (1) | - | 0 (1) |
| Romania | - | - | 0 (3) | - | - | 0 (24) | - | 0 (27) |
| Serbia | - | - | - | - | - | 0 (8) | - | 0 (8) |
| Slovakia | 0 (1) | - | 0 (3) | - | - | 0 (16) | - | 0 (20) |
| Slovenia | 0 (1) | - | 0 (1) | - | - | 1 (29) | - | 1 (31) |
| Spain | - | - | 0 (2) | 0 (1) | - | 13 (69) | - | 13 (72) |
| Sweden | 0 (1) | - | 1 (2) | 0 (1) | - | 29 (91) | 1 (1) | 31 (96) |
| Switzerland | 0 (1) | - | 0 (1) | - | - | 1 (132) | - | 1 (134) |
| Türkiye | - | - | 0 (2) | - | - | - | - | 0 (2) |
| United Kingdom | 1 (44) | - | 12 (145) | - | - | 74 (437) | - | 87 (626) |
| **Total** | **4 (307)** | **0 (3)** | **21 (993)** | **15 (44)** | **0 (3)** | **464 (4,066)** | **3 (3)** | **507 (5,419)** |

During the summer months of these years, considerably higher number of HPAI virus detections than those observed in previous epidemiological years were reported, giving a clear indication of persistent virus circulation. In contrast, and as of 1 September 2023, less than 40% of HPAI virus detections in domestic birds have been reported in the current compared to the previous epidemiological year (996 vs 2,537) (Figure 1b, Table 1). Similarly to previous years, a marked drop (close to zero) in the number of infected poultry establishments has been observed during the summer months.

Considering the current reporting period, from 24 June to 1 September 2023, 507 HPAI virus detections were reported in poultry (21), captive (4) and wild birds (482) (Table 1, Figure 2).

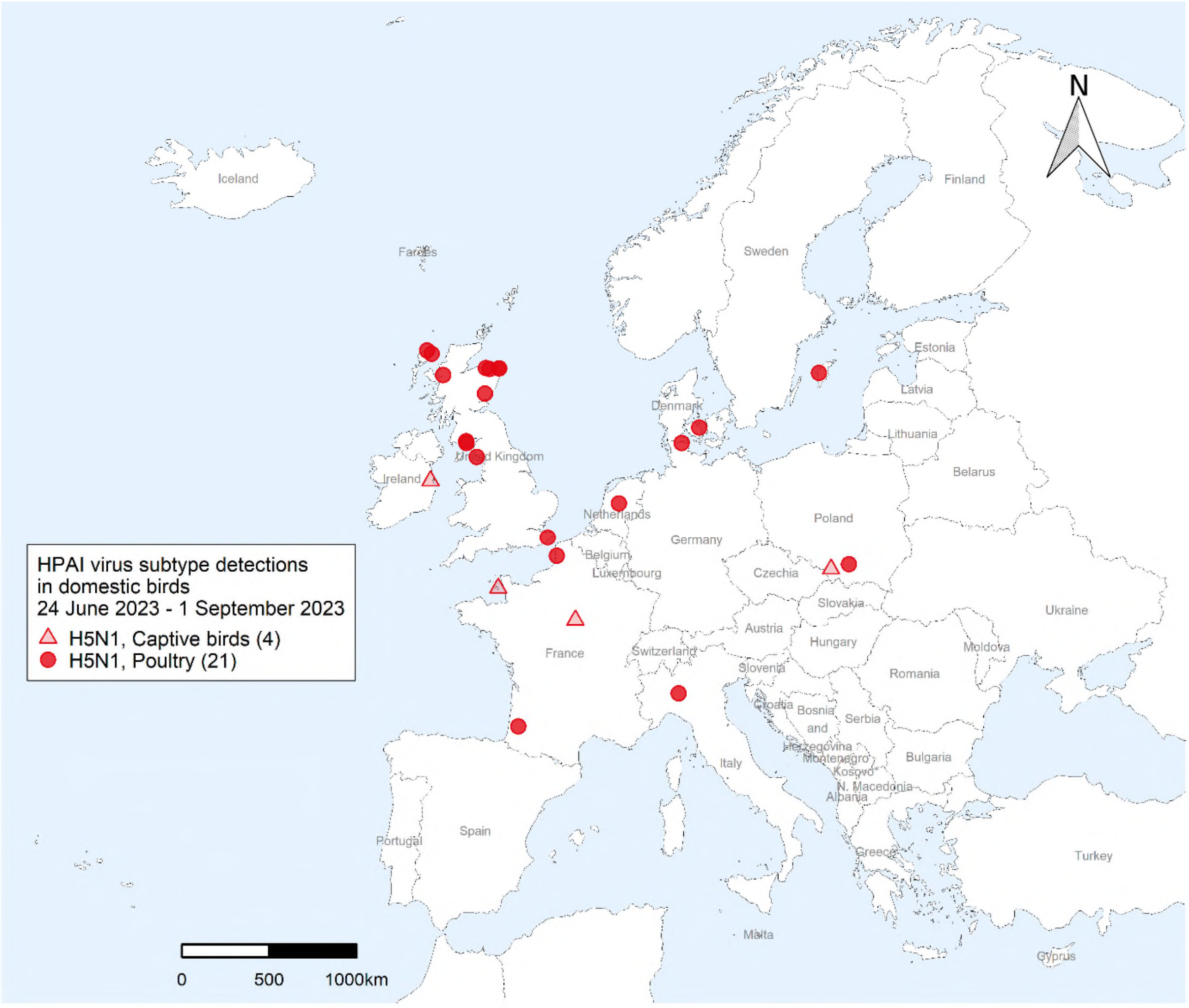

Author: EFSA
Data sources: ADIS, WOAH
Date updated: 01/09/2023

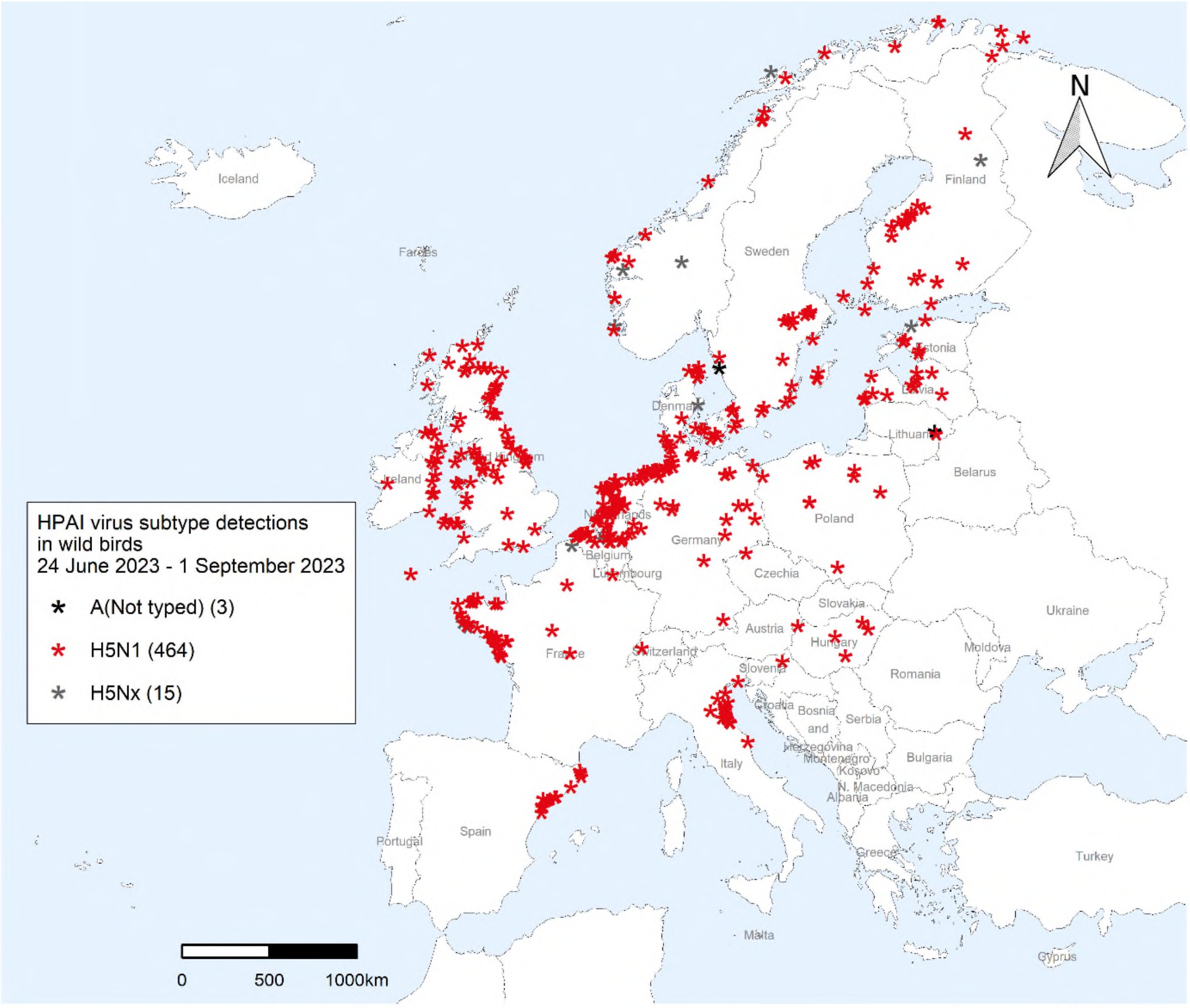

Author: EFSA
Data sources: ADIS, WOAH
Date updated: 01/09/2023

*This designation is without prejudice to positions on status, and is in line with United Nations Security Council Resolution 1244 and the International Court of Justice Opinion on the Kosovo Declaration of Independence.
United Kingdom data are from ADNS up to 31 December 2020. From 1 January 2021 onwards, the data source was WOAH for the United Kingdom (excluding Northern Ireland) and ADNS/ADIS for the United Kingdom (Northern Ireland)[5].
Source: ADIS, EFSA and WOAH (data extraction carried out on 1 September 2023).

**Figure 2:** Geographic distribution, based on available geocoordinates, of HPAI virus detections in poultry and captive birds (25) (upper panel), and in wild birds (482) (lower panel) reported by virus subtype in Europe from 24 June to 1 September 2023

HPAI outbreaks in both poultry and wild birds appear to be lined up along the coastlines of Europe (Figure 2). They overlap in several locations, such as along the coastlines of the United Kingdom (areas rich in seabird colonies), the Netherlands, Denmark and Sweden. Although only sporadic HPAI outbreaks in domestic birds have been reported during the summer months, spatially they are widely distributed in many European countries. Genetic analyses indicate that, in some instances, HPAI outbreaks in poultry are linked to seabirds (i.e. United Kingdom, France, Netherlands, Sweden). Many HPAI virus detections in wild birds are clustered in Northwest France and on the British Isles. Noteworthy is also a cluster of HPAI virus detections in black-headed gulls in the Southwest of Finland, which overlaps with HPAI outbreaks reported in farmed fur animals. Compared to the previous epidemiological year, there have been no new HPAI virus detections along the eastern

[5] In accordance with the Agreement on the Withdrawal of the United Kingdom from the EU, and in particular with the Protocol on IE/NI, the EU requirements on data sampling are also applicable to the United Kingdom (Northern Ireland).

Channel French coast, whereas several foci have appeared along the Mediterranean coastlines (i.e. Italy, Spain). Several HPAI virus detections in wild birds have also been reported inland, particularly in Germany, Hungary and Poland.

Following a peak at the end of May/beginning of June 2023 during the previous reporting period, mainly attributable to colony-breeding seabirds, there has been a progressive decline in the number of reported HPAI virus detections during the current reporting period to a relatively low number at the end of August (Figure 3). This decline coincides with the end of the breeding season of seabirds, after which they disperse from their colony sites. However, the actual number of HPAI virus detections in wild birds might even be higher due to the delay in outbreak reporting during the summer months and related holiday season.

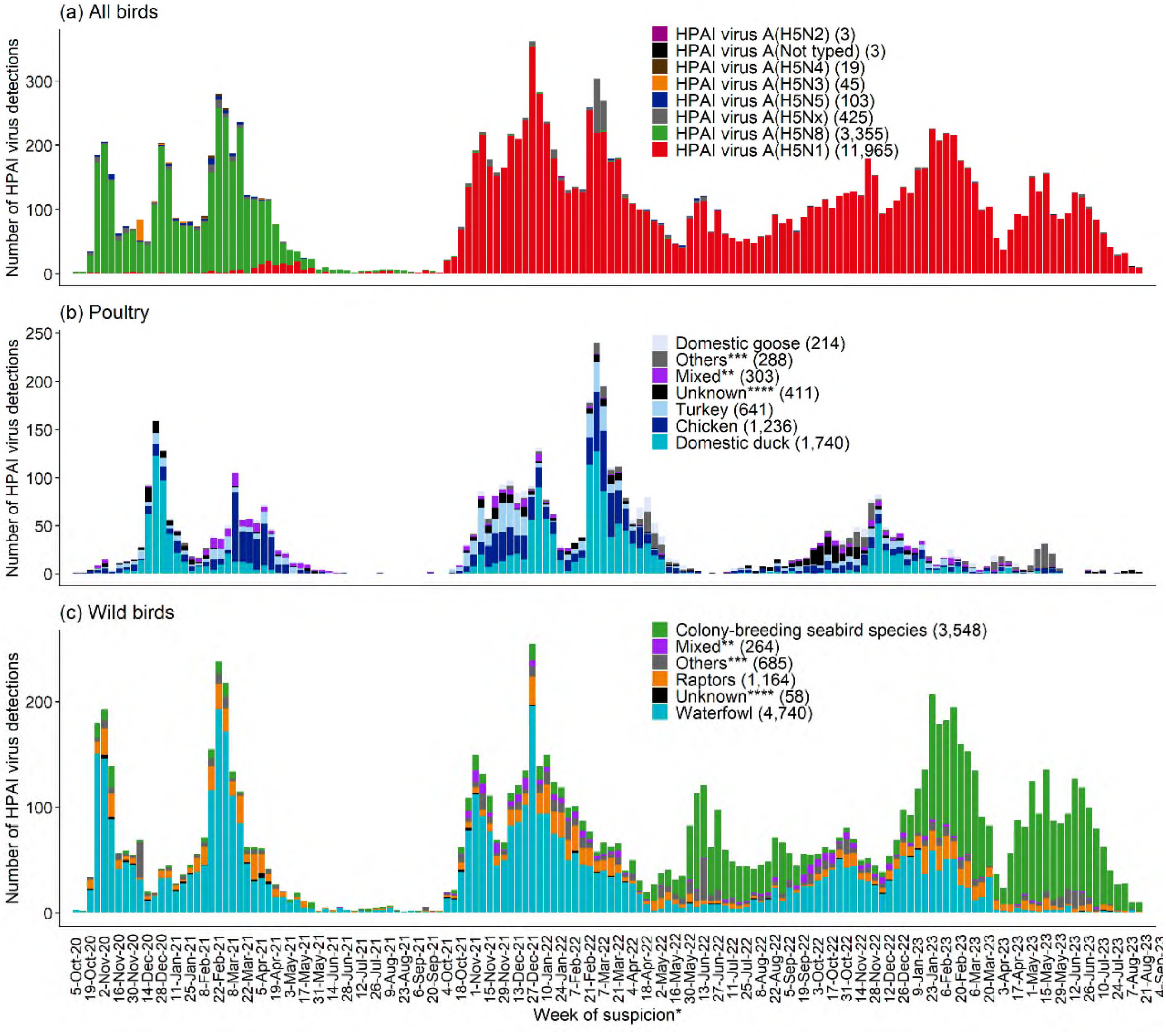

United Kingdom data are from ADNS up to 31 December 2020. From 1 January 2021 onwards, the data source was WOAH for the United Kingdom (excluding Northern Ireland) and ADNS/ADIS for the United Kingdom (Northern Ireland)[6].
*If the date of suspicion was not available, the date of confirmation was used to assign the week of suspicion.
**'Mixed' refers to outbreaks in which multiple species or categories were involved.
***'Others' groups all other affected categories that are not indicated in the legend.
****'Unknown' refers to affected categories that were not further specified during reporting.
Source: ADNS/ADIS, EFSA and WOAH (data extraction carried out on 1 September 2023).

**Figure 3:** Distribution of the total number of HPAI virus detections reported in Europe by week of suspicion (dates indicate the first day of the week) and virus subtype (15,918) (a),

[6] In accordance with the Agreement on the Withdrawal of the United Kingdom from the EU, and in particular with the Protocol on IE/NI, the EU requirements on data sampling are also applicable to the United Kingdom (Northern Ireland).

affected poultry categories (4,833) (b) and affected wild bird categories (10,459) (c), from October 2020 to 1 September 2023

The temporal pattern observed demonstrates the continuous presence of HPAI virus in the wild bird (seabird) population for the second consecutive summer. The comparatively low number of HPAI outbreaks in poultry during the same time period, however, suggests that few spillover events have taken place. In part, this might be explained by the fact that, except for certain gull species such as black-headed gulls, seabirds are less likely to visit poultry farm premises than waterfowl, in particular mallards (Elbers and Gonzales, 2020). The temporal correlation between the number of HPAI virus detections in waterfowl and poultry (Figure 3) may be also an indication of lower spillover risk from seabirds to poultry than from waterfowl to poultry.

Spatio-temporal information on all HPAI virus detections that have been reported in Europe since October 2016 is available via EFSA's interactive dashboard[7].

### Domestic birds

HPAI outbreaks in poultry were all identified as HPAI A(H5N1) and reported in the United Kingdom (12), France (3), Denmark (1), Germany (1), Italy (1), the Netherlands (1), Poland (1) and Sweden (1) (Table 1, Figure 4). Overall, more than 128,000 birds died or were culled in the HPAI virus-affected poultry establishments with the United Kingdom accounting for 59% of the birds that died or were culled (corresponding to 57% of the affected establishments), followed by France with 25% (Figure 4).

[7] http://hpai.efsa.aus.vet/

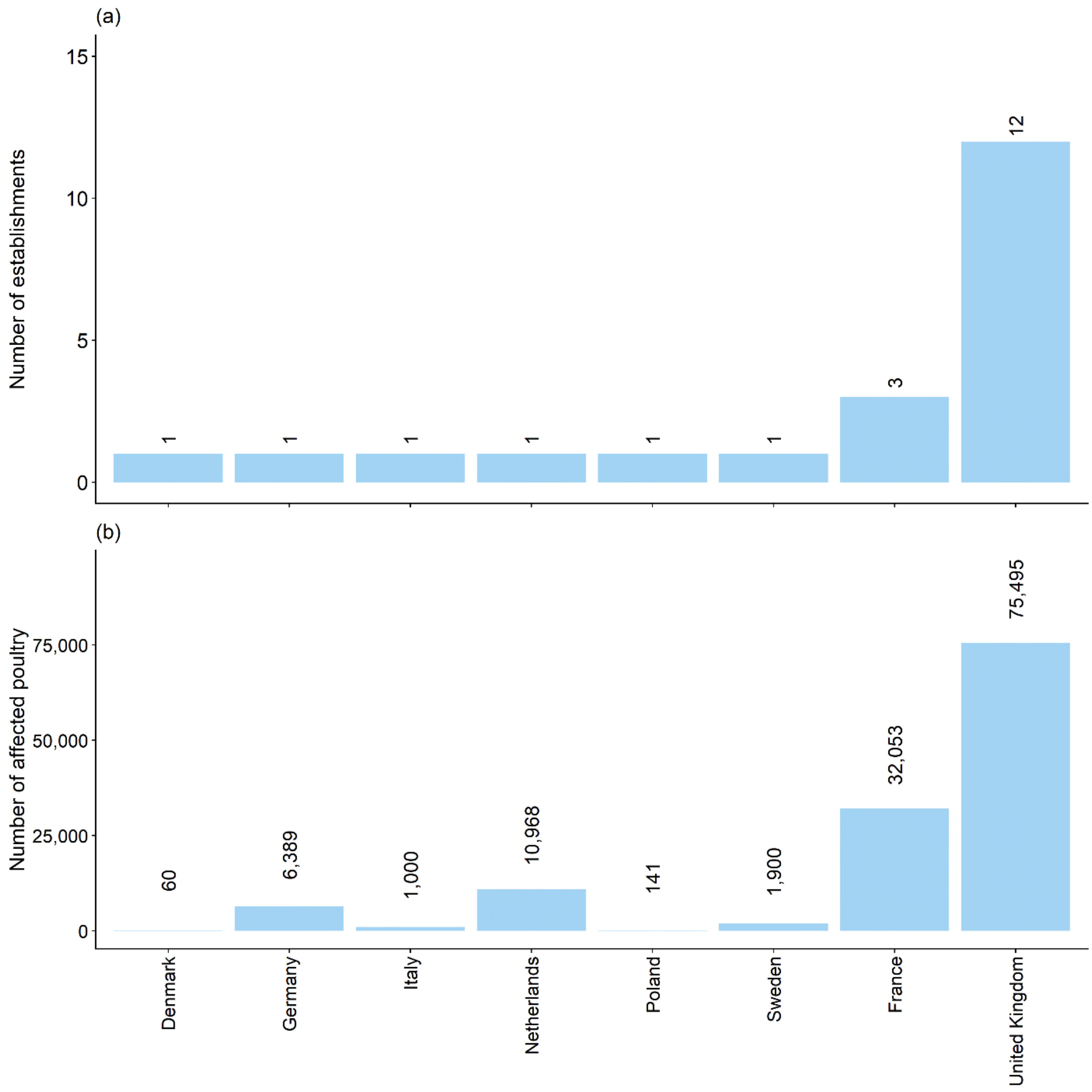

**Figure 4:** Number of HPAI virus-affected establishments (21) (a) and number of poultry in the HPAI virus-affected establishments (128,006) (b) in Europe between 24 June and 1 September 2023

Noteworthy is the relatively high number of HPAI outbreaks reported in the United Kingdom compared to continental Europe, where they appear to be sporadic and scattered across the continent. None of those primary outbreaks has led to secondary outbreaks during this reporting period. One of the HPAI outbreaks reported from France occurred on Réunion Island, along the West Asian-East African flyway.

In the following, a brief description of HPAI outbreaks in poultry is given by country. Information on the HPAI virus-affected establishments here reported was collected from the affected countries up to outbreaks that occurred by 18 August 2023.

Details on the characteristics of the affected poultry establishments and species reared are presented in Annex C.

*Denmark*

The primary outbreak reported occurred in a non-commercial farm housing chickens (n = 60 birds) that had outdoor access. Direct contact with wild birds was the most likely source of introduction. Increased mortality, presence of clinical signs as well as a drop in egg production and feed and water intake were reported. No data on the number of exposed people was available at the time of publication of this report.

*France*

During the current reporting period, three primary outbreaks were reported in poultry in France, one of them on Réunion Island at the beginning of July 2023. All the establishments were commercial farms: one of the two outbreaks detected on the French metropolitan territory was a mono-species farm keeping domestic ducks for foie gras production, where kept birds did not have outdoor access. No mortality or clinical signs were reported. The other outbreak was a multi-species farm rearing turkeys and chickens, for which information on outdoor access was not available. Only turkeys were reported to show clinical signs and a reduction in feed and water intake, with observed increased mortality; none of these observations were reported in chickens from the same poultry establishment. The outbreak reported on Réunion Island was a multi-species farm keeping chickens, domestic ducks and geese, for which information on outdoor access was not available. Increased mortality was reported among all 3 kept poultry species in the establishment. Information on the most likely source of introduction for these 3 outbreaks and the number of exposed people was unknown at the time of publication of this report.

*Germany*

One primary outbreak was reported in Germany. The outbreak occurred in a commercial farm keeping domestic geese for fattening, where birds did not have outdoor access. Increased mortality was reported and the most likely source of introduction was indirect contact with wild birds. No data on the number of exposed people was available at the time of publication of this report.

*Italy*

One primary outbreak was reported in Italy. The outbreak occurred in a commercial farm keeping pheasants for game purposes. The birds had outdoor access and the most likely source of introduction was indirect contact with wild birds. Increased mortality following nervous clinical signs was reported.

*Netherlands*

One primary outbreak was reported in the Netherlands. The establishment was a commercial farm keeping chickens for egg production. The farm reported increased mortality, decreased egg production, a drop in water and feed intake, and the presence of other clinical signs. The most likely source of introduction was unknown. No information on the number of exposed people was available at the time of publication of this report.

*Poland*

One primary outbreak was reported in Poland in a non-commercial farm (n = 141 birds). The establishment was a multi-species farm keeping chickens, domestic ducks and domestic geese. All birds had outdoor access. Unspecified clinical signs, increased mortality, and a drop in egg production as well as feed and water intake were reported in chickens, whereas none of the above-mentioned were reported in the other two poultry species. Indirect contact with wild birds was reported to be the most likely source of introduction after whole genome sequencing of the genetic material was carried out and compared with the virus collected from a white stork from Małopolskie Voivodeship, in which HPAI virus had been confirmed on 7 June 2023. Seventeen people were reported as exposed.

*Sweden*

A primary outbreak occurred in a commercial farm rearing chickens for egg production. The birds did not have outdoor access and the most likely source of introduction was indirect contact with wild birds, as there had been ongoing HPAI virus detections in wild birds in the area at the time the outbreak was reported. Increased mortality among chickens was reported as well as a drop in egg production and the presence of other clinical signs. The number of exposed people was 9.

HPAI virus detections in captive birds, all due to A(H5N1) viruses, were reported in France (1), Ireland (1, Chilean flamingo), Poland (1, ornamental hens) and the United Kingdom (1) (Figure 2, Table 1). The virus responsible for the outbreak in captive birds in Poland was genetically linked to those found in domestic cats during the previous reporting period.

### Wild birds

In contrast to the low number of HPAI outbreaks reported in poultry (except for the United Kingdom), there was a high number of HPAI virus detections in wild birds. A total of 464 A(H5N1), 15 A(H5Nx) and 3 A(Not typed) virus detections were reported in Germany (106), the United Kingdom (66), the Netherlands (59), France (36), Sweden (30), Norway (29), Belgium (25), Finland (24), Italy (22), Denmark (18), Spain (13), Latvia (13), Poland (9), the United Kingdom (Northern Ireland) (8), Ireland (8), Estonia (6), Hungary (4), Lithuania (2), Austria (1), Czechia (1), Slovenia (1) and Switzerland (1) (Figure 2, Figure 5, Table 1). The geographic distribution of reported HPAI virus detections in wild birds was still widespread, from north (north coast of Norway) to south (east-central Spain), and from northeast (Finland and Baltic states) to west (Ireland) (Figure 2, Figure 5, Table 1). Foci of reported HPAI virus detections were centred in the Wadden Sea region (Netherlands, Germany, Denmark), Northwest France and on the British Isles. These foci consisted mainly of colony-breeding seabirds, co-localised with smaller numbers of raptors (e.g. Netherlands and the Veneto region in Italy) and waterfowl.

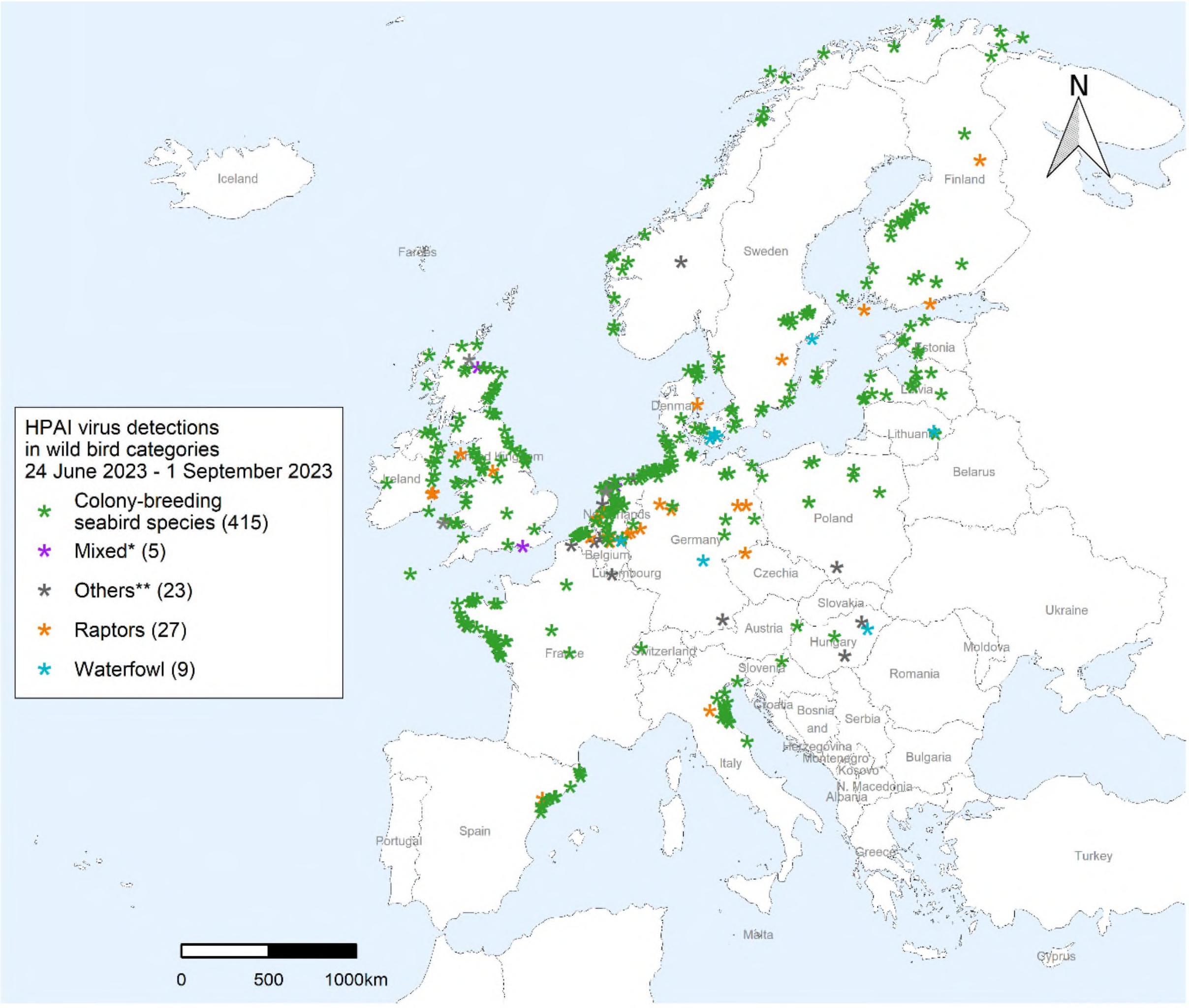

Author: EFSA
Data sources: ADIS, WOAH
Date updated: 01/09/2023

[*]This designation is without prejudice to positions on status, and is in line with United Nations Security Council Resolution 1244 and the International Court of Justice Opinion on the Kosovo Declaration of Independence.
Note that the unit reported is the number of HPAI virus detections in different wild bird species and not the number of HPAI virus detections in wild birds (as more than one species can be involved in one single HPAI virus detection reported).
[*]'Mixed' refers to outbreaks in which multiple categories were involved.
[**]'Others' groups all other affected categories that are not indicated in the legend.
Source: ADIS, EFSA and WOAH (data extraction carried out on 1 September 2023).

**Figure 5:** Geographic distribution, based on available geocoordinates, of HPAI virus detections in different categories of wild birds in Europe, by species category, from 24 June to 1 September 2023

The overall number of HPAI virus detections in wild birds (including detections in mixed wild bird species) (482) was 42% lower than the one of the previous reporting period (827 from 29 April to 23 June 2023), due to a lower number of HPAI virus detections affecting single wild bird categories: colony-breeding seabirds (415 vs 695), raptors (27 vs 43), waterfowl (9 vs 22), and other wild bird species (26 vs 62) (Figure 5). The same number of HPAI virus detections were reported in mixed outbreaks in the current and previous reporting periods (5). As observed during the last couple of months, seabirds continued to be affected in higher numbers than any other wild bird category, which is expected during summer when their population size is doubled and many naïve juveniles contribute to an amplification of the epidemic. This has been particularly true for gulls and terns (family Laridae, 406 of the 531 individual wild bird species affected), and, to a lesser extent, Alcidae (38) and Sulidae

(9) (Figure A.1 in Annex A, Figure 6). Compared to the previous reporting period, less Procellariidae species were involved (1 vs 25) with only northern fulmar reported as infected.

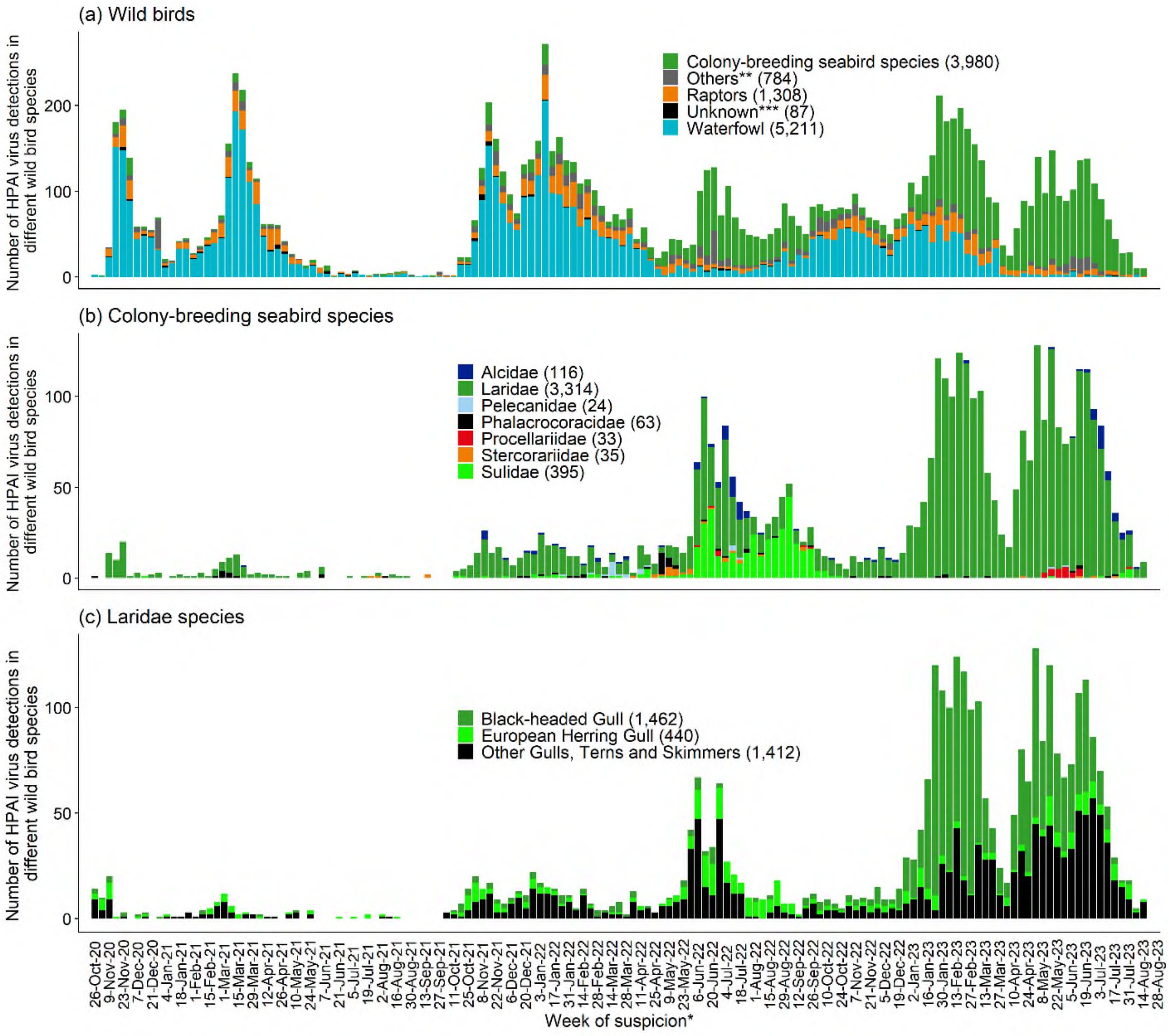

*If the date of suspicion was not available, the date of confirmation was used to assign the week of suspicion.
**'Others' contain unknown species or categories different from those displayed. The complete list of species by each wild bird category is reported in Table A.2 in Annex A.
***'Unknown' refers to affected categories that were not further specified during reporting.
Note that the scale of the vertical axes is specific to each panel and that the unit reported is the number of HPAI virus detections in different wild bird species and not the number of HPAI virus detections in wild birds (as more than one species can be involved in one single HPAI virus detection reported).
United Kingdom data are from ADNS up to 31 December 2020. From 1 January 2021 onwards, the data source was WOAH for the United Kingdom (excluding Northern Ireland) and ADNS/ADIS for the United Kingdom (Northern Ireland)[8].
Source: ADNS/ADIS, EFSA and WOAH (data extraction carried out on 1 September 2023).

**Figure 6:** Distribution of the total number of HPAI virus detections reported in Europe by week of suspicion (dates indicate the first day of the week) and (a) affected wild bird categories (11,370), (b) affected colony-breeding seabird families (3,980), and (c) affected Laridae species (3,314), from October 2020 to 1 September 2023

The top seven colony-breeding seabirds identified to species in which HPAI virus detections were reported were black-headed gull (111 in the current vs 378 in the previous reporting period), common tern (53 vs 64), European herring gull (50 vs 47), black-legged

[8] In accordance with the Agreement on the Withdrawal of the United Kingdom from the EU, and in particular with the Protocol on IE/NI, the EU requirements on data sampling are also applicable to the United Kingdom (Northern Ireland).

kittiwake (28 vs 13), sandwich tern (28 vs 20), common murre (27 vs 3) and Mediterranean gull (12 vs 11) (Figure A.1 and Figure A.2 in Annex A). The peak of HPAI virus detections from May to July corresponds to the breeding period of those species, and was followed by a rapid decline in HPAI virus detections corresponding to the dispersal of those species from their colony sites. The number (percentage) of colony-breeding seabirds identified to species in the current reporting period was equal (352/458 (77%)) to the one of the previous reporting period (578/754 (77%)). Laridae species identification remained difficult due to the difficulties in differentiating juvenile birds. HPAI A(H5N1) virus caused mass mortality in at least two new species, common murres and black-legged kittiwakes. Mass mortality of common murres, for example, was observed in Germany, where over 400 young-of-the-year were found at the base of breeding cliffs on Helgoland, and HPAI A(H5N1) virus was detected in 5 carcasses (NDR, online). In Vadsø, Norway, there was mortality of 15,000 black-legged kittiwakes over a three-week period in July and August associated with HPAI A(H5N1) virus (BirdLife International, online). In the United Kingdom, there was a mass die-off of common murres and black-legged kittiwakes associated with HPAI A(H5N1) virus in July (DEFRA, online).

The top three raptors identified to species in which HPAI virus detections were reported during this reporting period were peregrine falcon (11 in the current vs 17 in the previous reporting period), red kite (3 vs 1) and white-tailed eagle (3 vs 2) (Figure A.1 and Figure A.2 in Annex A).

In waterfowl, most of the HPAI virus detections were reported in mute swan (4 vs 3), and most of them sporadically occurred inland (e.g. Germany, Hungary) (Figure A.1 and Figure A.2 in Annex A).

The complete list of wild bird species found as HPAI virus-infected from 24 June to 1 September 2023 is reported in Figure A.1 in Annex A. The number of HPAI virus-affected wild birds that were not identified to species was 127/531 (24%), a slight improvement compared to the previous reporting period (242/888, 27%) (Figure A.1 in Annex A). In addition, HPAI virus detections in wild birds are generally underestimated and the numbers provided are not representative of the number of wild birds that actually died from A(H5) infection.

Note that Figures 5 and 6, as well as Figure A.1 in Annex A, provide information on the numbers of wild bird categories/families/species that have been detected as HPAI virus infected at single bird level, as more than one bird can be involved in one single reported HPAI virus detection.

### Prevention and control measures

A total of 27 countries provided information on prevention and control measures implemented as a consequence of HPAI outbreaks in poultry, wild birds or mammals in their own or neighbouring countries. Some of them did not apply any specific prevention or control measures during this reporting period. The complete description of prevention and control measures as reported by those countries up until 30 August 2023 is available in Annex B, whereas a summary of the information collected is provided below.

Several countries decided to implement or extend their national regulations regarding avian influenza as well as to reinforce measures in place concerning increasing awareness of avian influenza, biosecurity, adapting the restocking strategy, regulating hunting and recreational activities, and surveillance. Some of these measures are reported in detail below.

*Increasing awareness of avian influenza*

Most of the countries reported applying measures to increase awareness among both stakeholders and the general public following the outbreaks reported in wild birds and mammals. Information, guidelines and updates on the epidemiological situation were shared through press releases, official websites and social networks. For example, citizens have been able to notify the findings of dead wild birds to the Danish Veterinary and Food Administration (DVFA) through a mobile phone application called 'FugleinfluenzaTip'. Similarly, findings of dead wild birds (as well as dead wildlife in general) can be reported to the Swedish National Veterinary Institute (SVA) through the web application Rapporteravilt.sva.se. Latvia and Norway reported to provide recommendations and information campaigns for the general public on how to react in case of finding dead wild birds. Norway has a specific system in place to report dead wild birds.

Trainings for poultry keepers have been organised by Poland and are currently in place in Sweden.

Bulgaria implemented the national control and eradication programme for avian influenza, enhancing passive surveillance in wild birds.

*Housing order*

In Switzerland, an ordinance of the Federal Food Safety and Veterinary Office (FSVO) in force from 25 May until 15 October 2023 to prevent the spread of avian influenza prescribes vigilance measures for the whole country and control measures in case of HPAI in wild birds, stating that poultry within a radius of 1 km from a case in wild birds should be kept indoors for a minimum of 21 days. Italy amended and extended the ministerial provision on the housing order for poultry and on biosecurity.

Belgium reported that, after the improvement of the epidemiological situation in the country, from 10 June 2023 the housing order on poultry and birds from professional poultry holdings (registered establishments) was lifted. Since 25 April 2023, the housing order in southern regions of Sweden is no longer applied. Croatia amended the housing order in May 2023 to allow keeping of all poultry and captive birds in closed confinements in which contact with wild birds is prevented. In Ireland, regulations that required the confinement of poultry and captive birds, introduced in November 2022, were lifted on 18 April 2023. Considering the epidemiological situation, since August 2023, partial confinement measures were applied in some regions in the Netherlands. Denmark reported that the overall risk of infection with HPAI for poultry flocks due to contact with wild birds was assessed as 'medium' by the DVFA, and no housing order is enforced nor ban of gatherings is implemented currently. In Iceland, a housing order is still in place since March 2022. In Italy, the measures issued by the Ministry of Health provided for the confinement of poultry and captive birds in the areas at highest risk and the intensification of surveillance in poultry farms and wild birds, and in wild and domestic carnivore mammals. The housing order in a poultry dense region in south-western Norway was lifted on 31 May 2023, and no housing order is currently in place in Norway. In Cyprus, keeping backyard poultry in closed confinements is compulsory. In Estonia, keeping domestic birds outdoors is prohibited, unless poultry are protected from contact with wild birds.

*Biosecurity*

Poland intensified the controls on biosecurity, whereas other countries (Austria, Czechia, Finland, France, Greece, Netherlands, Slovakia, Spain) implemented or strengthened biosecurity measures for poultry in the whole country or in infected zones.

Finland implemented additional measures to be applied in the infected zones that were established following outbreaks in gulls in several locations, limiting the visits to poultry housing facilities in those zones and improving biosecurity at the entrance of farms (changing of footwear, hand washing and disinfection).

The Dutch government concluded agreements with suppliers who are able to deliver cleaning and disinfection equipment 24/7 within 4 hours after calling, following a culling order in any location across the country.

Italy established the systematic adoption of biosecurity measures according to the new Decree of 30 May 2023 'Application methods of biosecurity measures on poultry farms'.

Some limitations to the numbers and types of animal species kept in farms were applied: Italy required that dogs and cats housed in poultry premises should not have direct access to areas where poultry are kept, and Cyprus prohibited keeping ducks and geese together with other poultry species.

Greece reported that, following the HPAI outbreaks in American mink in Spain, public health and veterinary authorities, after consultation with experts, assessed the existing measures in place for Covid-19 in mink establishments (e.g. mandatory notification of increased animal morbidity/mortality, biosecurity measures including the use of FFP2 masks) as adequate to be applied for HPAI in farmed minks and humans in close contact with them.

*Adapting restocking strategy*

France reduced the density of duck farms to prevent the occurrence of a new wave of infections before the introduction of vaccination. Since 3 July 2023 and until the end of September, restocking poultry establishments with ducklings has been forbidden in 45 localities in western France (targeting areas with the highest densities of establishments involved in duck production) or around specifically listed strategic establishments in the same region: this corresponds to around 230 production units for fattening ducks (140,000 ducks per week) and other production units for foie gras production accounting for slightly less than 35,000 ducks per week.

Austria reported that, due to the current epidemiological situation in Austria and neighbouring countries, the whole country had been defined as a 'high-risk area'.

The Italian Ministry of Health has required that restocking of fattening turkeys in high-risk zones should be carried out using criteria established for homogeneous areas.

*Regulating hunting and recreational activities*

Different countries provided information in relation to their hunting policy. In Latvia, hunting of wild birds is permitted under the condition of respecting certain biosecurity measures. In Lithuania, hunting of waterbirds was forbidden by the Ministry of Environment until 15 August 2023. The Finnish Food Authority recommends avoiding hunting of waterfowl in areas or places where avian influenza has been detected or mass deaths of wild birds occurred.

Since mid-August, with the start of the hunting season, Bulgaria has set up training campaigns for hunters to increase awareness of HPAI prevention, surveillance and control.

Due to a mass mortality event among black-legged kittiwakes, Norway banned the access to three nature protection areas in northern Norway with vulnerable wild bird species colonies and requested the public not to walk in areas with a high density of wild birds.

*Surveillance*

As mentioned previously, Greece assessed the mandatory notification of increased animal morbidity/mortality in place in mink establishments as adequate.

#### 2.1.2 HPAI virus detections in birds outside Europe

An overview of the HPAI virus detections in birds that were notified from other countries not reporting via ADIS but via WOAH from 24 June to 1 September 2023 is presented in Table 2 and Figure 7.

**Table 2:** Number of HPAI virus detections in non-European countries, by virus subtype and country, from 24 June to 1 September 2023

| Region | Country | Domestic birds | | Wild birds | | | Total |
|---|---|---|---|---|---|---|---|
| | | A(H5N1) | A (Not typed) | A(H5N1) | A(H5Nx) | A (Not typed) | |
| Africa (31) | Nigeria | 1 | - | - | - | - | 1 |
| | South Africa | - | 27 | - | - | 2 | 29 |
| Americas (50) | Argentina | 1 | - | - | - | - | 1 |
| | Brazil | 1 | - | 34 | - | - | 35 |
| | Chile | 2 | - | 2 | - | - | 4 |
| | Colombia | - | - | - | - | 2 | 2 |
| | Costa Rica | - | - | - | 2 | - | 2 |
| | Ecuador | 3 | - | - | - | - | 3 |
| | United States of America | 3 | - | - | - | - | 3 |
| Asia (4) | China | - | - | 1 | - | - | 1 |
| | Taiwan | 3 | - | - | - | - | 3 |
| Europe (15) | Russia | 4 | - | 11 | - | - | 15 |
| **Total** | | **18** | **27** | **48** | **2** | **4** | **99** |

'-' means that no HPAI outbreaks were notified to WOAH.
Source: WOAH (data extraction carried out on 1 September 2023).

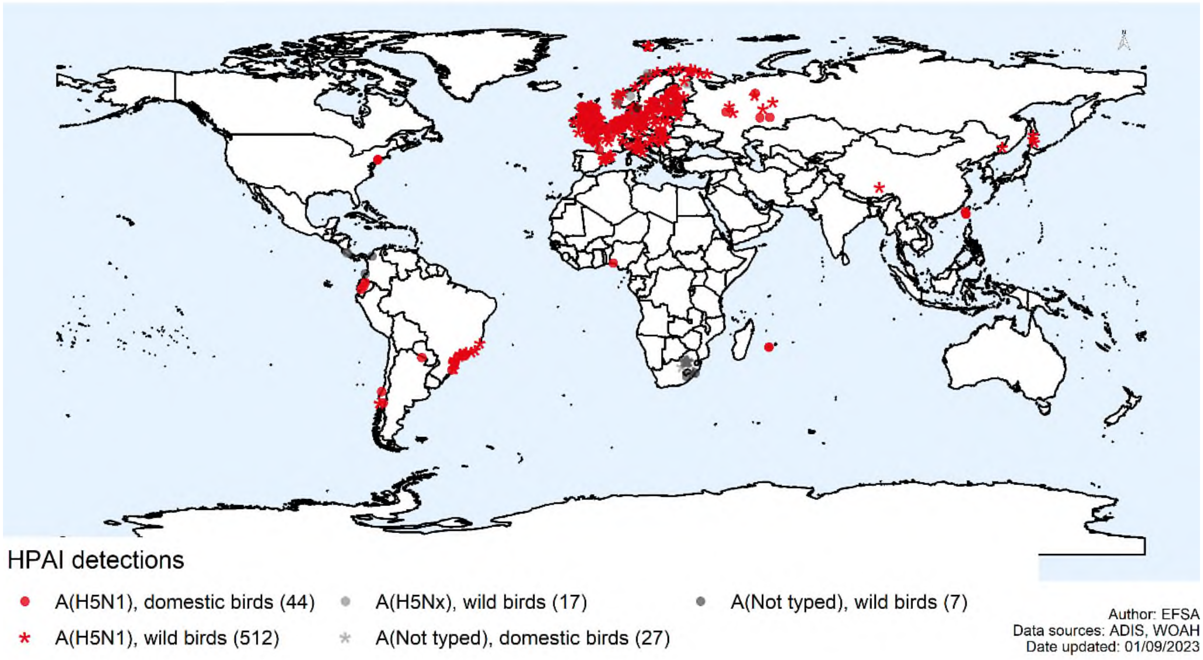

**Figure 7:** Geographic distribution, based on available geocoordinates, of HPAI virus detections reported worldwide in domestic birds (71) and wild birds (536) by virus type, from 24 June to 1 September 2023

Worldwide, HPAI A(H5) virus continued to be detected in poultry and wild birds and reported to WOAH from a slightly higher number of affected countries (12 vs 11) compared to the previous reporting period from 29 April to 23 June 2023 (EFSA, ECDC and EURL, 2023). In the Americas, the number of affected countries has remained stable, but in contrast to the last report, Canada, Paraguay and Uruguay did not report any further A(H5) outbreaks, while Colombia, Costa Rica and Ecuador again detected A(H5) virus during this reporting period. In Africa and Asia, the number of affected countries increased with reports from Nigeria, China and Taiwan, but no additional outbreaks were reported from Nepal. Overall, outbreaks in poultry increased significantly (45 vs 35), but conversely, fewer cases in wild birds were reported to WOAH (54 vs 86) in the current reporting period. Therefore, less HPAI virus detections in poultry and wild birds were notified to WOAH overall (99 vs 121).

Nigeria reported an outbreak of HPAI A(H5N1) on a large poultry farm on the southern coastline in July 2023. The outbreak of A(H5) in South Africa continued on large to very large poultry farms. Media reports have mentioned that HPAI A(H7N6) virus has spread through Mpumalanga, Gauteng and the Free State over the past couple of months alongside with the more common H5N1 virus strain, resulting in a great impact on the poultry industry (FAO, online; ProMed, online-a). Furthermore, A(H5) virus was detected in several wild bird species, such as greater crested tern, pied crow and grey crowned crane. In Asia, outbreaks of A(H5N1) in poultry have only been reported from Taiwan, occurring on large farms of chickens, ducks and quails. China reported an outbreak of HPAI A(H5N1) in brown-headed gulls and other wild birds in Nagqu City, in the Tibet Autonomous Region (Avian Flu Diary, online-a). Russia detected HPAI A(H5N1) virus in two backyard and two very large poultry farms as well as in many wild birds of the families Laridae and Alcidae in the western and far eastern part of the country. Genetic analysis suggests that HPAI viruses identified in Laridae in the Leningrad region were introduced from Europe. The situation in Chile and the United States of America continued to be similar in terms of number of outbreaks in poultry compared to the previous reporting period. Furthermore, Chile detected HPAI A(H5N1) virus in South American tern, Guanay cormorant and Peruvian booby. Argentina notified only one outbreak of HPAI A(H5N1) on a small backyard farm to WOAH. In addition to Argentina, also Ecuador only reported outbreaks in poultry but no wild bird cases during the current reporting period. Brown pelicans infected with HPAI virus have been found sick and/or dead in Costa Rica (Cahuita national park (FluTrackers, online)) and Colombia (Gorgona national park (Minuto30, online)). The large outbreak in wild birds on the Brazilian Atlantic coast continued, and several species, such as royal tern, Cabot's tern, brown-hooded gull, and southern lapwing, including also the Magellanic penguin, were notified to WOAH. Furthermore, Brazil officially reported the detection of one HPAI A(H5N1) outbreak on a small backyard farm in the same region. The actual situation in the Americas is continuously evolving with new HPAI virus detections in wild and domestic birds as well as in mammal species. With the onset of spring in the southern hemisphere, the risk increases that HPAI virus will spread to seabirds and pinnipeds in the Antarctic in the near future (OFFLU, online).

The list of wild bird species that have been reported to WOAH as HPAI virus-infected from 24 June to 1 September 2023 is presented in Table A.1 in Annex A.

In the tables and figures of the current report, only data extracted from WOAH on 1 September 2023 are presented. However, A(H5) virus detections in poultry and wild birds are also reported to the public via different means. This additional information about HPAI virus detections that was available from sources other than WOAH-WAHIS has not been systematically retrieved, and is described in the below text.

In general, official ministry and local veterinary authority websites as well as social media report a higher number of outbreaks than notified to WOAH. In particular, in Brazil, the number of outbreaks in poultry and wild birds is reported to be significantly higher (ND Mais, online; Liberal, online) so that in some federal states, such as Espírito Santo, and nationwide, an animal health emergency for 180 days has been declared (Folha Machadense, online). The veterinary authorities of Uruguay confirmed the first cases of A(H5) not only in sea lions but also in penguins (Montevideo Portal, online). Furthermore, Ecuador detected HPAI A(H5N1) virus in wild birds on the Galapagos islands for the first time. Dead birds were found on several islands and three samples were tested positive (Parque Nacional Galápagos, online). Outside of the Americas, reports of outbreaks of HPAI A(H5N1) in poultry in Indonesia and Togo are under review at the Food and Agriculture Organization of the United Nations and WOAH, respectively (WOAH-WAHIS, online; ProMed-b, online).

#### 2.1.3 Genetic characteristics of HPAI viruses of the A(H5N1) subtype circulating in Europe in avian species

Details on the nomenclature of the HPAI A(H5) viruses used in this section are reported in Appendix B.

**Genetic diversity of HPAI A(H5N1) viruses in avian species**

The vast majority (> 95%) of the genetically characterised HPAI A(H5N1) viruses (> 1,750) collected since October 2022 from 24 European countries belong to three genotypes, namely AB (H5N1 A/duck/Saratov/29-02/2021-like), CH (H5N1-A/Mallard/Netherlands/18/2022-like) and BB (H5N1-A/Herring_gull/France/22P015977/2022-like). Since February 2023, the BB genotype has become the most frequently identified variant that reached a frequency ranging from 83 to 100% in the period April–August 2023 (based on the data available up to 12 September 2023).

Genotype BB has been detected mainly in Laridae, with the black-headed gull representing the most affected species during the ongoing epidemic. During the past winter season it had mainly circulated in West and South Europe, whereas since spring 2023 it has spread eastward and northward, showing a wide geographic expansion.

In domestic birds, AB and CH have been the most frequently detected genotypes in the 2022–2023 epidemiological year. However, starting from February 2023, the number of poultry outbreaks associated with the BB genotype has increased and during the summer months (June–July 2023), this genotype was responsible for most of the outbreaks, based on the available data. Outbreaks reported in domestic birds not associated with the BB genotype were identified in Italy, where a new genotype was characterised, and in Poland, where the infection was caused by a virus highly related to the viruses identified in cats in the country (genotype CH).

**Mutations identified in HPAI A(H5N1) viruses from avian species**

Molecular analyses of the HPAI A(H5N1) viruses circulating in birds in Europe during the 2022–2023 epidemiological year indicate that they continue to be well-adapted to avian species, as they retain a preferential binding for avian-like receptors. However, several mutations, previously described in literature (Suttie et al., 2019), have been identified as being associated with i) enhanced polymerase activity and replication in mammals or mammalian cells, ii) increased virulence, iii) increased/conferred resistance towards antiviral drugs, iv) increased in vitro binding to human-type receptors alpha2,6-SA, v) decreased antiviral response in ferrets, vi) evasion of human butyrophilin subfamily 3 member A3 (BTN3A3), and vii) disruption of the second SIA-binding site, with a frequency varying by the distinct mutations. The real effect of these mutations on the biological characteristics of the viruses is still unknown and further studies are needed to improve existing knowledge. Among the identified mutations, molecular markers associated with an increased replication and/or virulence in mammals (PB2-E627K, PB2-D701N, PB2-T271A) have been rarely (0.4%) detected in viruses from wild (n = 3, with PB2-E627K) and domestic (n = 4: two PB2-E627K, one PB2-D701N, one PB2-T271A) birds collected in different European countries since October 2022, as observed in the previous epidemiological years. Among the mutations in the HA protein that have proved to increase in vitro binding to human-type receptors, some of them (i.e. S133A, S154N, T156A, H5 numbering) have been identified in the majority of the HPAI A(H5N1) viruses circulating in Europe since October 2022, while others (i.e. D94N, S155N, V210I) have only been sporadically observed. The impact of these HA mutations on the biological characteristics of the circulating viruses is still unknown; however, none of them have been demonstrated to cause a shift from avian-like to human-like receptor binding preference. Moreover, almost all the HPAI A(H5N1) viruses belonging to the BB genotype (H5N1 A/gull/France/22P015977/2022-like) contain mutations NP-Y52N (Pinto et al., 2023) and NA-S369I (Du et al., 2018), which may increase their zoonotic potential. Mutations associated with antiviral resistance have been occasionally identified in the circulating strains. Specifically, about 3% of the characterised viruses contain mutations associated with reduced inhibition by neuraminidase inhibitors (NAIs) and 1.7% possess mutations which can cause increased resistance to amantadine and rimantadine.

### 2.2 HPAI virus detections in non-human mammals

#### 2.2.1 HPAI virus detections in non-human mammals worldwide

From 24 June 2023 to 15 September 2023, HPAI A(H5N1) and A(H5Nx) viruses were reported in farmed, pet and wild mammals around the world. Data were collected from WOAH-WAHIS and supplemented by additional information provided by Member States and media reports.

In farmed mammals, during this reporting period, A(H5N1) outbreaks were reported in 26 fur animal farms in Finland. In all those farms the animals were kept in wire cages and had outdoor access, with varying level of contact to wild animals, such as birds and rodents, and the most likely source of introduction was direct contact with wild birds. Among the farms with a confirmed infection, there are 18 farms with only foxes, three with foxes and American minks, three with foxes and common raccoon dogs, one with foxes, American minks and common raccoon dogs and one farm with only common raccoon dogs. The size of affected farms varied between 700 and 50,000 animals. Twenty-one out of 23 farms keeping Arctic foxes reported increased mortality among the animals (for two farms the information was not available), while the presence of clinical signs was reported in 17 farms. This information was unknown or not available for the remaining farms at the time of publication of this report. Both farms keeping American minks reported an increased mortality among the animals, the presence of clinical signs was observed only in one of the farms, whereas this information was not available for the other ones. Increased mortality was reported in two farms keeping common raccoon dogs, clinical signs among animals were reported in one of the farms, whereas information was not available for the other ones. Sequencing results obtained from the affected farms showed both the possibility of animal-to-animal transmission after a single introduction of the virus and potential between-farm transmission. Preliminary results from serological tests carried out on Arctic foxes and American minks that had contact with positive animals suggest that these species may be affected without showing clinical signs or mortality. However, these findings are not conclusive and will require further epidemiological and molecular evidence to support them. All minks on A(H5N1)-positive farms were culled and, starting from 11 September 2023, Finland has adopted a new culling policy and established the culling of all animals on HPAI virus-positive farms, including foxes and common raccoon dogs (Ruokavirasto, online). In China, a novel reassortant A(H5N6) virus has been detected in farmed dogs (Yao et al., 2023).

In pet mammals, three cats in Seoul, the Republic of Korea, were found infected with HPAI A(H5N1) virus at the end of July 2023 (Aju Daily Korea English, online; The Korea Herald, online). Two of those cats were co-living in an animal shelter in Yongsan-gu (25 July), where < 35 cats had been dying for several weeks after showing respiratory and neurological signs, and another one was living in an animal shelter in Gwanak-gu. The latter cat was presented to an animal hospital for anorexia and respiratory symptoms, but it died during the following days of treatment. Emergency quarantine measures involving cleaning and disinfection, access control and epidemiological investigations were implemented. Susceptible animal breeding facilities within a 10 km radius were put under surveillance and exposed people were inspected. Of note, the HPAI A(H5N1) virus strain identified in cats in the Republic of Korea differs from the one identified in cats in Poland.

In wild mammals, in Europe, HPAI virus detections continued in red fox in Finland, Latvia, Norway, Sweden and the United Kingdom. Some of those animals were still puppies, some showed neurological signs, and the red fox affected in the United Kingdom represented the first mammal case in Northern Ireland. A Eurasian otter was found infected in Finland. In Germany, a harbour seal in a rehabilitation centre was found positive for A(H5N1) virus, while six other harbour seals in direct contact with the animal remained unaffected. It was reported that gulls (European herring gulls and lesser black-backed gull) would regularly fly into the facility during feeding times. The affected harbour seal showed clinical signs and a drop in feed and water intake before it died. More infected harbour seals were reported from Denmark (Avian Flu Diary, online-b) and the United States of America, for the first time on the West coast of the country, in Washington State (NOAA, online). In Russia and Argentina, the northern fur seal and southern elephant seal (infobae, online) were found positive for A(H5N1) virus for the first time. Outside Europe, most cases in mammals were reported from South America. HPAI A(H5N1) and A(H5Nx) outbreaks continued in south American sea lion in Argentina and Uruguay, and in South American fur seal in Uruguay. Of note, Uruguay holds the largest reserve of South American sea lions on the east coast of Latin America. Many of those HPAI outbreaks in seals occurred in areas with large penguin colonies. The detection of this virus in a South American sea lion at Puerto Williams, Chile (54.9 S), in June 2023, and in 7 of 21 South American sea lions found dead in Río Grande, Argentina (53.5 S), are the southernmost detections of the virus to date. There is a substantial risk that it will continue southwards and in the near future reach Antarctica and its offshore islands, which are home to more than 100 million breeding birds, six species of pinnipeds and 17 species of cetaceans. This risk may be increased in coming months due to the spring migration of wild birds from South America to breeding sites in the Antarctic. The negative impact of HPAI A(H5) on Antarctic wild birds and mammal populations could be immense, both because of their likely susceptibility to mortality from this virus, and their occurrence in dense colonies of up to thousands of pinnipeds and hundreds of thousands of birds, allowing efficient virus transmission (OFFLU, online).

Although not occurred during this reporting period, Canada reported the detection of HPAI A(H5N5) virus in red fox in April 2023, and Germany reported the first case of HPAI A(H5N1) in a European pine marten back in April 2022. All other HPAI virus detections in mammals that occurred before 29 April, but were only reported during the current reporting period, are included in Table 3.

**Table 2:** Avian influenza A(H5Nx) virus detections in mammal species other than humans related to circulating viruses worldwide, 2016–2023

| Virus | Animal (order, family, species) | | | Country | Reference |
|---|---|---|---|---|---|
| A(H5N1) or A(H5Nx) clade 2.3.4.4b | Artiodactyla | *Suidae* | Domestic pig (*Sus scrofa*)* | Italy | Rosone et al. (2023) |
| | Carnivora | *Canidae* | Arctic fox (*Vulpes lagopus*) | Finland | WOAH |
| | | | Bush dog (*Speothos venaticus venaticus*) | United Kingdom | WOAH |
| | | | Common raccoon dog (*Nyctereutes procyonoides*) | Finland, Japan | WOAH |
| | | | Coyote (*Canis latrans*) | United States of America | WOAH |
| | | | Dog (*Canis lupus familiaris*) | Canada, Italy* | WOAH<br>Moreno et al. (2023) |
| | | | Japanese raccoon dog (*Nyctereutes viverrinus*) | Japan | FAO |
| | | | Red fox (*Vulpes vulpes*) | Belgium, Canada, Denmark, Estonia, Finland, France, Germany, Ireland, Italy, Japan, Latvia, Netherlands, Norway, United Kingdom (Northern Ireland), United Kingdom, United States of America, Sweden | WOAH, SVA<br>SSI (online-a), USDA (online)<br>Personal communication by Māra Užule (Latvia)<br>Personal communication by Susan Cusack (United Kingdom (Northern Ireland)) |
| | | *Felidae* | Amur leopard (*Panthera pardus*) | United States of America | USDA (online) |
| | | | Amur tiger (*Panthera tigris*)** | United States of America | WOAH |
| | | | Bobcat (*Lynx rufus*) | United States of America | WOAH |
| | | | Caracal (*Caracal caracal*) | Poland | Personal communication by Krzysztof Jażdżewski (Poland) |
| | | | Cat (*Felis catus*) | Canada, France, Italy*, Korea (Rep. of), Poland, United States of America | WOAH<br>Briand et al. (2023) |
| | | | Lion (*Panthera tigris*) | Peru | WOAH |
| | | | Lynx (*Lynx lynx*) | Finland | FFA (online) |
| | | | Mountain lion (*Puma concolor*) | United States of America | WOAH |
| | | *Mephitidae* | Striped skunk (*Mephitis mephitis*) | Canada, United States of America | WOAH<br>CTV News (online), USDA (online) |
| | | *Mustelidae* | American mink (*Neovison vison*) | Canada, Spain | WOAH<br>Xunta de Galicia (online) |
| | | | Beech marten (*Martes foina*) | Netherlands | GISAID (online) |
| | | | Eurasian otter (*Lutra lutra*) | Netherlands, Finland | WUR |
| | | | European badger (*Meles meles*) | Netherlands | WUR |

| Virus | Animal (order, family, species) | | | Country | Reference |
|---|---|---|---|---|---|
| | | | European pine marten (*Martes martes*) | Germany | WOAH |
| | | | European polecat (*Mustela putorius*) | Belgium, Netherlands | WUR |
| | | | Ferret (*Mustela furo*) | Belgium, Slovenia | WOAH |
| | | | Fisher cat (*Pekania pennanti*) | United States of America | WOAH |
| | | | Marine otter (*Lontra felina*) | Chile | WOAH |
| | | | Mountain lion (*Puma concolor*) | United States of America | WOAH |
| | | | North American River otter (*Lontra canadensis*) | United States of America | WOAH |
| | | | Southern river otter (*Lontra provocax*) | Chile | WOAH |
| | | *Otariidae* | Northern fur seal (*Callorhinus ursinus*) | Russia | WOAH |
| | | | South American fur seal (*Arctocephalus australis*) | Peru, Uruguay | FAO |
| | | | South American sea lion (*Otaria flavescens*) | Argentina, Chile, Peru, Uruguay | WOAH |
| | | *Phocidae* | Caspian seal (*Pusa caspica*) | Russia | FAO |
| | | | Grey seal (*Halichoerus grypus*) | Canada, Germany, Netherlands, Poland, United Kingdom, United States of America | WOAH<br>AMMI (2022), Monica et al. (2023) |
| | | | Harbour seal (*Phoca vitulina*) | Canada, Denmark, Germany, United Kingdom, United States of America | WOAH<br>Agriland (online) |
| | | | Southern elephant seal (*Mirounga leonina*) | Argentina | infobae (online) |
| | | *Procyonidae* | Raccoon (*Procyon lotor*) | Canada, United States of America | WOAH |
| | | | South American coati (*Nasua nasua*) | Germany, Uruguay | WOAH |
| | | *Ursidae* | American black bear (*Ursus americanus*) | Canada, United States of America | WOAH<br>Healthy Wildlife (online), KTOO (online) |
| | | | Asian black bear (*Ursus thibetanus*) | France | GISAID (online) |
| | | | Brown bear (*Ursus arctos*) | United States of America | Kiniradio (online), USDA (online) |
| | | | Kodiak grizzly bear (*Ursus arctos horribilis*) | United States of America | FAO |
| | Cetacea | *Delphinidae* | Bottlenose dolphin (*Tursiops truncatus*) | Peru, United States of America | WOAH<br>UFHealth (online) |
| | | | Chilean dolphin (*Cephalorhynchus eutropia*) | Chile | FAO<br>Sernapesa (online) |
| | | | Common dolphin (*Delphinus delphis*) | Peru, United Kingdom | WOAH |

| Virus | Animal (order, family, species) | | | Country | Reference |
|---|---|---|---|---|---|
| | | | | | Leguia et al. (2023) |
| | | | White-sided dolphin (*Lagenorhynchus acutus*) | Canada | Avian Flu Diary (online-c) |
| | | *Phocoenidae* | Burmeister's porpoise (*Phocoena spinipinnis*) | Chile | FAO |
| | | | Harbour porpoise (*Phocoena phocoena*) | Sweden, United Kingdom | SVA |
| | Didelphimorphia | *Didelphidae* | Virginia opossum (*Didelphis virginiana*) | United States of America | WOAH<br>USDA (online) |
| A(H5N5) clade 2.3.4.4b | Carnivora | *Canidae* | Red fox (*Vulpes vulpes*) | Canada | WOAH |
| | | *Procyonidae* | Raccoon (*Procyon lotor*) | Canada | WOAH |
| A(H5N6) clade 2.3.4.4b | Carnivora | *Canidae* | Dog (*Canis lupus familiaris*) | China | Yao et al. (2023) |
| A(H5N8) clade 2.3.4.4b | Artiodactyla | *Suidae* | Domestic pig (*Sus scrofa*)* | France | Hervé et al. (2021) |
| | | | Wild boar (*Sus scrofa*)* | Germany | Schülein et al. (2021) |
| | Carnivora | *Canidae* | Red fox (*Vulpes vulpes*) | United Kingdom | Floyd et al. (2021) |
| | | *Phocidae* | Grey seal (*Halichoerus grypus*) | Poland, Sweden, United Kingdom | SVA<br>Shin et al. (2019), Floyd et al. (2021)<br>Personal communication by Siamak Zohari (Sweden) |
| | | | Harbour seal (*Phoca vitulina*) | Denmark, Germany, United Kingdom | Floyd et al. (2021), Ärzteblatt (online), Avian Flu Diary (online-d), Outbreak News (online), SSI (online-b–c) |

*Serological detection.

#### 2.2.2 Genetic characteristics of HPAI viruses of the A(H5N1) subtype circulating in Europe in non-human mammals

Details on the nomenclature of the HPAI A(H5) viruses used in this section are reported in Appendix B.

**Genetic diversity of HPAI A(H5N1) viruses in non-human mammals in Europe**

Since October 2020, the complete genome sequences (n > 230) of HPAI A(H5) viruses of clade 2.3.4.4b collected in 15 European countries from 17 distinct mammalian species (i.e. European badger, Asian black bear, bush dog, cat, South American coati, ferret, red fox, Arctic fox, Eurasian lynx, American mink, Eurasian otter, European polecat, harbour porpoise, harbour seal, grey seal, beech marten, caracal and common raccoon dog) have been generated. The characterised viruses belong to 8 different HPAI A(H5N1) and A(H5N8) genotypes previously identified in birds. During the 2022–2023 epidemiological year, most of the mammalian infections were caused by the three most widespread genotypes in birds, namely BB (H5N1-A/gull/France/22P015977/2022-like), AB (H5N1-A/duck/Saratov/29-02/2021-like) and CH (H5N1-A/Eurasian_Wigeon/Netherlands/3/2022-like).

The characterised A(H5N1) viruses infecting the mammalian species identified in Europe in the summer months (June–August 2023) belong to the following genotypes: i) CH, which affected domestic cats in Poland, ii) BB, which was identified in wild red foxes in Finland and Norway as well as in fur animal farms in Finland, and iii) AB, which was detected in a harbour seal in Denmark from an outbreak involving several seals. The virus from this last case was related to viruses identified in deceased mute swans found nearby.

About 47% of the characterised viruses contain at least one of the adaptive markers associated with an increased virulence and replication in mammals in the PB2 protein (E627K, D701N, T271A or K526R) (Suttie et al., 2019). These mutations have rarely been identified in the HPAI A(H5) viruses of clade 2.3.4.4b collected in birds in Europe since October 2020. This observation suggests that these mutations with potential public health implications have likely emerged upon transmission to mammals.

Outside Europe, it is worth mentioning that in April 2023 an A(H5N6) virus belonging to clade 2.3.4.4b was identified in farmed dogs in China. The characterised virus contains the Q226L (H3 numbering) mutation in the receptor binding site of the HA protein, which represents one key mutation for the switch in the binding specificity from avian-type to human-type receptor (Yao et al., 2023).

*HPAI A(H5N1) viruses in cats in Poland*

Thirty-one HPAI A(H5N1) viruses belonging to the CH genotype were collected in domestic cats (n = 30) and in one caracal from eight Voivodeships in Poland between the second half of June and July 2023 (Domańska-Blicharz et al., 2023; Rabalski et al., 2023). As described in the previous report (EFSA, ECDC and EURL, 2023), the viruses are highly related and cluster with a virus of the same genotype detected in the country in a white stork at the beginning of June and in a backyard chicken farm at the end of June. A virus with high degree of similarity to the cat viruses was found in chicken meat from the same household as an ill cat, suggesting, but not confirming, a possible route of transmission (Domańska-Blicharz et al., 2023; Rabalski et al., 2023). This genotype has widely been circulating in birds in Europe since October 2022. In Poland, between December 2022 and January 2023, it was responsible for several outbreaks in poultry, mainly in the Wielkopolskie Voivodeship, as well as for cases in wild birds. Since February 2023, this genotype has only been sporadically identified in the country.

All the HPAI A(H5N1) viruses detected in domestic cats and in a backyard chicken farm in Poland in mid June–July 2023 possess two mutations in the PB2 protein, E627K and K526R, which are molecular markers of virus adaptation in mammals (Suttie et al., 2019;

Domańska-Blicharz et al., 2023; Rabalski et al., 2023). To date, the Polish A(H5N1) viruses are the only 2.3.4.4b viruses showing both mutations. Of note, the PB2-E627K substitution was also present in the A(H5N1) virus detected in the white stork at the beginning of June.

*HPAI A(H5N1) viruses in fur animal farms in Finland*

HPAI A(H5N1) viruses (> 90) collected in July and August 2023 from Arctic foxes, red foxes, American minks and common raccoon dogs from 24 fur animal farms in Finland were genetically characterised. All viruses belong to genotype BB and are highly related to each other and with the viruses collected from gulls in the same area (Lindh et al., 2023). In some farms, a possible within-farm virus evolution has been observed, while in other farms genetic data suggest the occurrence of multiple virus introductions.

Critical mutations associated to virus adaptation to mammalian species (PB2-E627K and PB2-T271A) have been observed in viruses collected from five farms. Moreover, besides mutation NA-369I in the second sialic acid binding site (2SBS), which is typical of all the viruses of the BB genotype, viruses collected from one farm contain a second mutation in the 2SBS, NA-396V. The 2SBS, which specifically binds to alpha 2,3-linked sialic acid receptors, is conserved in most avian viruses but has mutated and consequently lost in human viruses as well as in viruses from some other hosts (Du et al., 2018, 2021). Moreover, HPAI A(H5N1) viruses collected from 17 different infected farms possess mutation M2-S31N, which confers an increased resistance to amantadine and rimantadine. This same mutation has been identified in several A(H5N1) viruses collected from gulls in the same geographic area.

### 2.3 Avian influenza virus infections in humans

#### 2.3.1 Most recent human infections with avian influenza A(H3N8), A(H5N1), A(H5N6) and A(H9N2) viruses

Since the last report and as of 28 September 2023, seven new human cases or detections with avian influenza infection were reported from the United Kingdom (two cases A(H5N1)) and China (three A(H5N6), two A(H9N2)) (Table 4). All seven people had exposure to poultry or live poultry markets prior to avian influenza virus detection or onset of illness.

**Table 4:** Most recent human cases due to avian influenza viruses, by virus subtype

| Subtype | New cases reported | Cases/detections in 2023 | Total cases (deaths) | Countries reporting human cases |
|---|---|---|---|---|
| A(H3N8) | - | 1 case | 3 (1)<br>Since 2022 | China |
| A(H5N1) | 2 new detections in the United Kingdom | 8 cases/detections | 878 (458)<br>Since 2004 | 23 countries reported cases;<br>EU/EEA: Spain with virus fragment detections in two poultry workers considered contamination and no productive infections |
| A(H5N6) | 3 new cases in China | 4 cases | 88 (34)<br>Since 2014 | China (87), Laos (1) |

| | | | | |
|---|---|---|---|---|
| A(H9N2) | 2 new cases in China, including one case with no neuraminidase identified | 5 A(H9N2) cases, 1 A(H9Nx) case | 127 (2) Since 1998 | No EU/EEA country; China (111), Egypt (4), Bangladesh (3), Cambodia (2), Oman (1), Pakistan (1), India (1), Senegal (1) |

Source: ECDC line list and WHO.

#### 2.3.2 Human A(H3N8) cases, summary

No new cases have been reported since the previous report and as of 14 September 2023. During 2022 and 2023, in total three infections have been reported from China with two in children (4 and 5 years old) and one in an adult (56-year-old female) exposed to either infected backyard poultry or live poultry markets. The infections were mild in one case and more severe or critical in the other two (Bao et al., 2022; Sit et al., 2022). Human-to-human transmission has not been reported and all three cases were considered sporadic spill-over events.

#### 2.3.3 Human A(H5N1) cases, summary

The United Kingdom reported two new detections of A(H5N1) of clade 2.3.4.4.b (A/gull/France/22P015977/2022-like genotype) in two individuals who were involved in culling and cleaning activities in two different poultry farms affected with confirmed A(H5N1) virus outbreak among birds (GovUK, online-a). One of the detections is said to be difficult to interpret and 'may be consistent with infection or contamination of the respiratory tract', while the other detection is considered a contamination (GovUK, online-a; WHO, online). For the person with inconclusive infections/detection assessment, sore throat and myalgia were reported with no definitive association between time of positivity and symptoms. The other person did not report symptoms. The detections were identified through a surveillance study 'Asymptomatic avian influenza surveillance of poultry workers'.

In 2022, five countries reported six human cases/detections (Figure 8): China (1), Ecuador (1), Spain[9] (2), United States of America (1) and Vietnam (1). In 2023, four countries reported eight cases/detections: Cambodia (2, clade 2.3.2.1c), Chile (1), China (1) and the United Kingdom (4).

As of 14 September 2023, there have been 878 human cases including 458 deaths with A(H5N1) infection notified to the World Health Organization (WHO) from 23 countries (Azerbaijan, Bangladesh, Cambodia, Canada, Chile, China, Djibouti, Ecuador, Egypt, Indonesia, India, Iraq, Laos, Myanmar, Nepal, Nigeria, Pakistan, Spain[9], Thailand, Türkiye, Vietnam, United Kingdom, United States of America; Figure 8).

[9] A(H5N1) detections in two Spanish poultry workers involved in culling activities are considered contaminations and not productive infections.

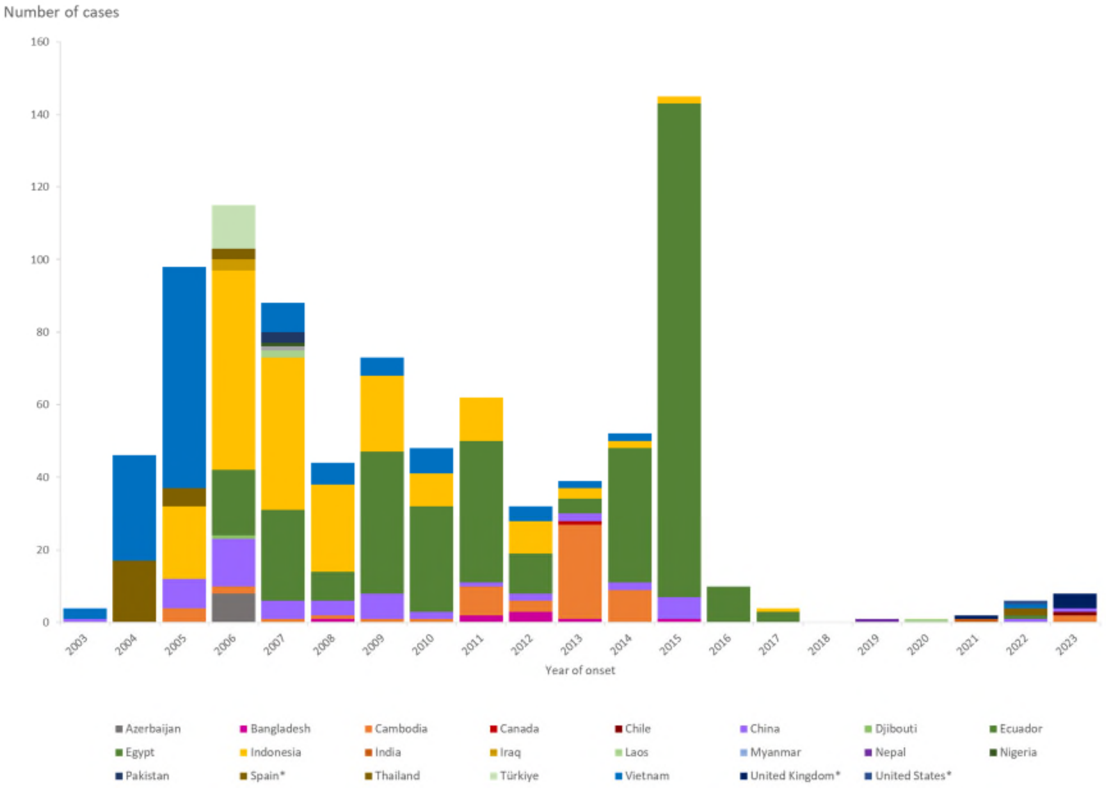

*Includes detections due to suspected environmental contamination and no evidence of infection reported in 2022 from Spain (2) and the United States (1), and in 2023 from the United Kingdom (3, 1 inconclusive).

**Figure 8:** Distribution of confirmed human cases of HPAI A(H5N1) virus infection by year of onset and country, 2003–2023 (data as of 14 September 2023, n = 878)

#### 2.3.4 Human A(H5N6) cases, summary

Since the last report, three additional cases have been reported from China in different provinces. A 27-year-old woman and a 64-year-old man were reported to be in critical conditions, a 68-year-old man died. All cases had exposure to dead poultry or backyard poultry before onset of symptoms (The Government of the Hong Kong Special Administrative Region, online).

As of 28 September 2023 and since 2014, China (87) and Laos (1) reported a total of 88 human infections with A(H5N6) (Figure 9), including 34 (39%) with fatal outcome according to notifications to WHO. Since 2021, the majority of A(H5N6) viruses identified in humans belong to clade 2.3.4.4b, although sequence information is not available for all viruses. After the modification of the H5 component in the vaccine used for poultry in China in January 2022, the number of human cases declined. However, with the available information it is not possible to make any further conclusions on whether the vaccination caused the observed decline (Chen et al., 2022; Cui et al., 2022; Gu et al., 2022; Zhu et al., 2022).

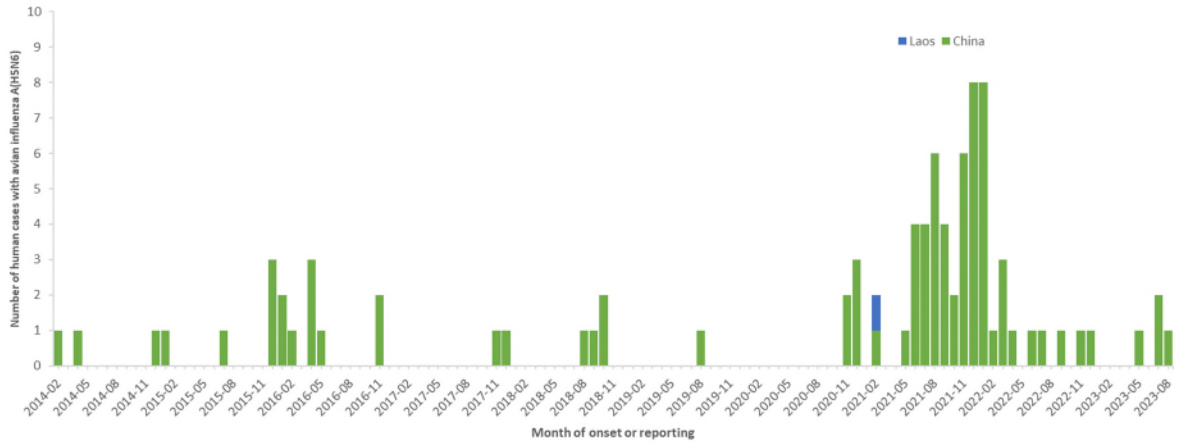

**Figure 9:** Distribution of confirmed human cases of A(H5N6) virus infection by month and year of onset and country, 2014–2023 (data as of 28 September 2023, n = 88)

#### 2.3.5 Human A(H9N2) cases, summary

Since the last report and as of 14 September 2023, two new human cases with A(H9) infection have been reported by China, one A(H9N2) and one A(H9) with pending neuraminidase determination (F.I.C., online). The A(H9N2) cases is a 59-year-old farmer who developed pneumonia following suspected exposure to backyard poultry. The A(H9) detection is in a 4-year-old girl.

As of 14 September 2023 and since 1998, a total of 127 human infections including two fatalities with A(H9N2) have been reported from eight countries all outside the EU (Figure 10): Bangladesh (3), China (114), Cambodia (2), Egypt (4), Pakistan (1), Oman (1), India (1) and Senegal (1). The majority of infections were identified in children up to 9 years of age (95; 76%) and women were more affected than men (73 vs 50; 2 of unknown sex).

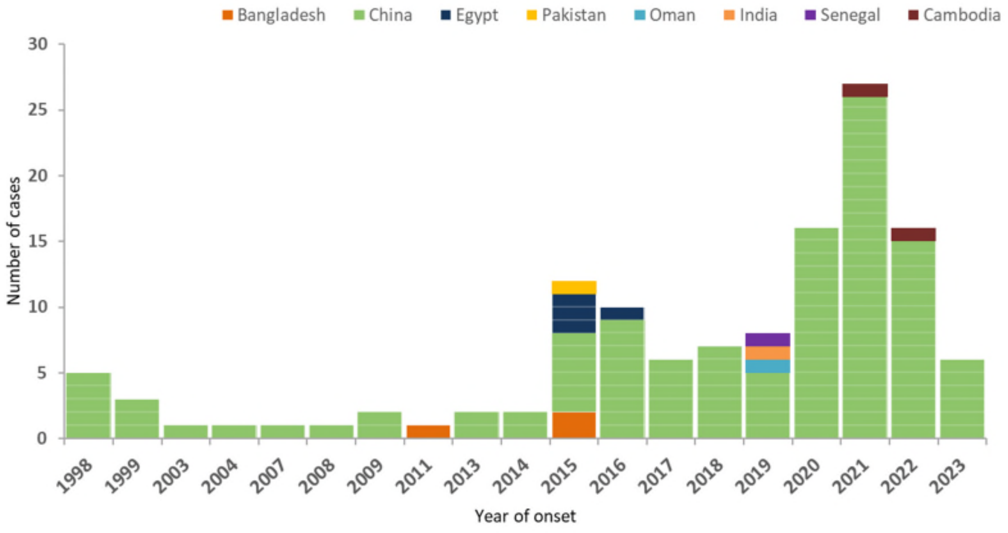

*One case due to H9 with missing NA typing result.

**Figure 10:** Distribution of confirmed human cases of avian influenza A(H9N2) virus infection by month and year of onset and country, 1998–2023* (as of 14 September 2023, n = 127)

#### 2.3.6 Genetic characteristics of HPAI viruses of the A(H5Nx) subtype from humans

Based on the data available from the GISAID's EpiFlu™ Database[10], since 2020 human infections have been caused by four different A(H5) clades of the A/goose/Guangdong/1/1996-lineage, namely 2.3.2.1c (Laos, 2020 and Cambodia, 2023), 2.3.2.1a (India, 2021), 2.3.4.4h (China, 2020–2021) and 2.3.4.4b (China, Europe, North America, South America, 2020–2023).

Mutation Q226L was identified in two A(H5N6) viruses of clade 2.3.4.4b collected in China in 2021 (Li et al., 2022; Zhu et al., 2022). This mutation is associated with the switch in the receptor specificity from avian-type to human-type receptor (Stevens et al., 2006; Chutinimitkul et al., 2010; Russell et al., 2012). This mutation was previously detected in two A(H5N1) viruses of clade 1 collected from human infections in Cambodia in 2013 (Rith et al., 2014) and, based on the available sequence data, it is not present in the clade 2.3.4.4b A(H5Nx) viruses currently circulating in the avian population in Europe. Moreover, six clade 2.3.4.4b A(H5N6) viruses collected from human infections in China in 2021 possessed one of the adaptive markers in the PB2 protein (Q591K, E627K or D701N) associated with an increased virulence and replication in mammals (Li et al., 2022; Zhu et al., 2022).

Available sequence information from the virus A/Chile/25945/2023 detected from the human case in Chile in March 2023 shows that the 2.3.4.4b clade virus carries two amino acid substitutions in PB2, indicative of mammalian adaptation, Q591K and D701N. Q591K has been associated with increased polymerase activity and replication in mammalian cell lines and increased virulence in mice, while D701N was also associated with increased virulence and contact transmission in guinea pigs (Li et al., 2005; Gao et al., 2009; Le et al., 2009; Steel et al., 2009; Yamada et al., 2010; Taft et al., 2015; Suttie et al., 2019).

#### 2.3.7 Additional information and international risk assessments

Different studies showed that currently circulating A(H5N1) viruses are able to replicate efficiently in human respiratory tract cell types and that in ferret infection and transmission studies, animals got infected but showed only very mild symptoms mostly limited to upper respiratory tract and one virus carrying the PB2-E627K mutation was efficiently transmitted through direct contact between ferrets, resulting in lethal outcomes (Pulit-Penaloza et al., 2022; Kobasa et al., 2023).

Challenges as to how to distinguish true productive infections with avian influenza from contaminations in settings with high viral load were described in a study from 2012/13 testing workers at live bird markets in Bangladesh, where influenza-like symptoms, PCR positivity and seroconversion have been studied (Hassan et al., 2023).

A study on the human-isolated avian A(H3N8) virus showed that this virus can replicate in human bronchial epithelial and lung epithelial cells as well as transmit between ferrets through the respiratory droplets also underlining the critical importance of PB2-E627K as mediator for airborne transmission (Sun et al., 2023). A(H3N8) viruses are low pathogenic and are widespread in chicken flocks in China. Isolates derived from humans had acquired human-receptor-binding preference caused severe pathology in mice and ferrets.

The previously issued risk assessment using the Influenza Risk Assessment Tool (IRAT) of the United States Centers for Disease Control and Prevention (US CDC) placed the risk of clade 2.3.4.4b viruses in the lower moderate category (CDC, 2021). The risk of the A(H5N1) clade 2.3.4.4b viruses currently circulating in the United States bird and poultry populations, which are closely related to European viruses, was assessed by the US CDC to be low for human health in the general population and higher for people occupationally or recreationally exposed to birds (CDC, online-a, c). An updated IRAT assessment for the

[10] https://gisaid.org/

A(H5N1) clade 2.3.4.4.b virus from the mink farm outbreak in Spain in 2022 scored slightly higher in some risk elements than the virus of clade 2.3.4.4.b previously assessed overall indicating a comparable 'moderate' risk level (CDC, online-b).

The WHO assessed the risk related to the recent A(H5N1) viruses infecting humans as low for the general public and low to moderate for occupationally exposed people (WHO, 2022). WHO previously assessed the risk for A(H5N6) as follows: 'the zoonotic threat remains elevated due to spread of the viruses in birds, based on evidence available so far, the overall pandemic risk is considered not significantly changed in comparison to previous years'. The UK Health Security Agency (UKHSA) assesses the situation to remain at level 3 (with limited mammalian transmission that excludes humans but with low confidence) on a scale ranging between 0–6 similar to the previous assessment (GovUK, online-b–d). Additional information is also available in the Joint ECDC/EFSA/EURL/EU-OSHA document on 'Testing and detection of zoonotic influenza virus infections in humans in the EU/EEA, and occupational safety and health measures for those exposed at work' (ECDC, 2022a).

#### 2.3.8 ECDC risk assessment

Overall, the risk of infection of humans with avian influenza viruses of the currently circulating clade 2.3.4.4b A(H5) virus in Europe for the general public in EU/EEA countries remains low.

The risk to occupationally or otherwise exposed groups to avian influenza infected birds or mammals including infected cats remains at low to moderate level. This assessment covers different situations that depend on the level of exposure.

More information on the methodology used for the assessment of the risk can be found at the published ECDC operational tool on rapid risk assessment methodology (ECDC, 2022b).

Viruses continue to spread and diversify globally causing outbreaks in poultry and wild birds as well as spillover events to different mammalian species. Reassortment events will likely continue globally leading to a more complex situation. Viruses currently circulating in bird populations in Europe are avian-adapted viruses, e.g. they bind to avian-like receptors, transmit and replicate best in bird species. However, mutations associated with mammalian adaptation have been identified sporadically in birds but more frequently in infected mammals. Despite the occurrence of mutations associated with mammalian adaptation, the viruses analysed retain preferential binding to avian receptors. Additional studies are required to better understand the impact of the different mutations that particularly evolved in the mammalian hosts. Various mutations associated with mammalian adaptation, which could increase the transmission to and replication in humans, adds challenges to the overall risk assessment.

Despite indications of mammal-to-mammal transmission e.g. between seals or farmed fur animals such as American mink, common raccoon dogs or foxes, up to now, no mammal-to-human transmission of clade 2.3.4.4b viruses has been observed. No transmission to exposed people has been identified during the investigation related to infected cats in Poland, nor has any human infection been detected in relation to the outbreaks in fur farms in Finland.

Avian influenza transmission from animals to humans is a rare event, nevertheless, transmission to humans cannot be excluded when people without protection are in direct contact with infected animals or contaminated environment. Despite large number of human exposure events to clade 2.3.4.4b viruses in EU/EEA over the last three years e.g. exposure to poultry during culling operations or wild bird findings, no symptomatic infection has been identified and reported.

Human infections with severe disease progression and fatal outcomes have been observed outside the EU and such severe infections can also not be excluded should infections in the EU occur. The majority of human cases developing symptoms after infection with clade 2.3.4.4b viruses were reported to have had unprotected exposure to infected sick or dead poultry mostly in backyard settings but also exposed to contaminated environment.

Based on the analyses of known molecular markers associated with resistance towards antiviral drugs in the available sequences, the majority of the circulating A(H5N1) viruses in Europe remain susceptible to licensed antivirals for use in seasonal influenza virus infections as well as for pandemic use (NAIs: oseltamivir, zanamivir), M2 blockers (adamantanes), and cap-dependent endonuclease blocker (baloxavir marboxil). Antivirals are available as treatment options and post-exposure prophylaxis. A higher number of viruses were resistant to NAI during the period covered and analysed in this report compared to earlier time periods. This requires some attention and in-depth analysis to better understand the occurrence but should also raise more attention to a potential emergence of NAI-resistant viruses circulating in wild birds and should be monitored more closely.

The proposed candidate vaccine viruses prepared for pandemic preparedness (to develop H5 vaccines for humans) are considered to be antigenically similar to the currently circulating clade 2.3.4.4b viruses in Europe. Vaccines against A(H5) viruses for the use in humans are not available at the moment. However, the only A(H5N1) vaccine available in the EU is currently being updated to include an AIV strain to match the circulating virus/clade and it is expected to be authorised before end of 2023.

For the upcoming winter period with the annual respiratory virus epidemic, ECDC suggests a risk-based targeted surveillance approach that focusses on outbreaks and severe respiratory or unexplained neurological disease in hospitalised patients:

- People admitted to hospitals with respiratory symptoms should be asked about exposure to sick or dead birds, wild or other animals in the two weeks prior to symptom onset or before admission (if symptom onset date cannot be defined) and should be tested according to an assessment of risk by the clinician. Specimens from hospitalised patients with very severe influenza virus infections could be considered for subtyping, particularly if believed to be part of a nosocomial outbreak.

- Hospitalised patients with unexplained viral encephalitis/meningoencephalitis should be considered for testing for seasonal influenza virus, and type A virus-positive specimens should be further subtyped for seasonal influenza viruses and therefore rule out avian influenza virus.

- Clusters of severe respiratory infections requiring hospitalisation should be investigated and testing for avian and other influenza viruses should be considered if routine testing for respiratory pathogens is inconclusive.

The new surveillance guidance for the influenza season 2023/34 'Targeted surveillance to identify human infections with avian influenza virus during the influenza season 2023/24, EU/EEA' is available here: https://www.ecdc.europa.eu/en/publications-data/avian-influenza-infections-surveillance-eu-eea

This document complements the earlier published guidance that covered the summer period 2023: Enhanced surveillance of severe avian influenza virus infections in hospital settings in the EU/EEA (europa.eu)

## 3. Conclusions

### 3.1 Birds

- During this reporting period, sporadic HPAI A(H5N1) outbreaks in domestic birds, all of them primary, occurred throughout Europe and mainly in the United Kingdom. Most of these outbreaks seemed to be lined up along the coastlines of the British Isles and Central Europe. No particular production system was more affected than others. Primary introduction via wild birds (mainly seabirds, and most likely gulls) can be suspected, as the viruses detected in poultry in almost all cases belonged to the BB genotype, which is currently widespread in wild birds (apart from Italy and Poland). In contrast to these outbreaks, the virus isolated from a Polish backyard poultry farm appears to be related to the viruses previously identified in a white stork and in cats in Poland (cases described in the previous report). Genetic analyses revealed that the outbreak in Italy was due to a completely new genotype (reassortant). The absence of secondary spread after primary introduction in the EU may be due to the location of infected poultry premises in low farm density areas, and/or due to the different infectivity of the BB genotype in poultry.

- Outside Europe, HPAI A(H5N1) virus continued to spread mostly in South America and there is a substantial risk that it could expand to Antarctica in the near future. Overall, fewer HPAI virus detections in poultry and wild birds were reported by a similar number of countries outside Europe to WOAH compared to the previous reporting period. In particular, although outbreaks in poultry increased significantly, the cases in wild birds decreased even more.

- It has become more difficult than in previous years to predict the epidemiology of HPAI A(H5N1) in Europe in the coming weeks because of its presence in two categories of wild birds, colony-breeding seabirds and waterfowl. On the one hand, virus prevalence in black-headed gulls and other seabird species may continue to decline following dispersal of those birds from their colony sites. On the other hand, virus prevalence in waterfowl may increase over the autumn migration as those animals, including naïve young-of-the-year, arrive at wintering areas in Europe. However, this increase may be moderated by the presumed higher levels of flock immunity in these wild bird populations. This expectation can be further supported by the observed decreasing trend in the numbers of HPAI virus detections reported in waterfowl since the 2020–2021 epidemiological year. Overall, this may result in a moderate increase in HPAI outbreaks on poultry farms in the coming weeks. Given the current geographic and temporal pattern of HPAI virus detections in wild birds in Europe and higher numbers compared to the same period in the previous year, the detections in wild birds in the current epidemiological year have already surpassed the number of detections reported in 2021–2022.

- Besides black-headed gulls and common terns, which were the most affected wild birds species during this reporting period, HPAI A(H5N1) virus also caused mass mortality in two wild bird species that were previously only rarely affected: common murres (mainly in the United Kingdom) and black-legged kittiwakes (mainly in the United Kingdom and Norway). The absence of a coordinated European surveillance system for wild bird mortality does not allow to provide a complete picture of the impact of HPAI virus on wild birds in Europe.

- A slight improvement, compared to the previous reporting period, was observed in the capability of identifying the HPAI virus-infected wild birds at species level; however, for Laridae species, the inability to identify the species in juvenile birds remains challenging.

- The HPAI A(H5N1) viruses currently circulating in Europe retain a preferential binding for avian-like receptors; however, several mutations associated with increased zoonotic potential have been detected. Their effects on the biological characteristics of the viruses need to be further investigated.

### 3.2 Mammals

- Wild and domestic carnivores continued to be the most affected mammalian species by HPAI viruses.
- During this reporting period, HPAI virus-infected domestic carnivores included pets and farmed fur animals. Infected cats and dogs were reported outside Europe (Republic of Korea and China), while Finland reported 26 HPAI A(H5N1) outbreaks in fur animal farms keeping American mink, red and Arctic fox, and common racoon dog. The animals were kept in wire cages and had outdoor access with the most likely source of infection being direct contact with gulls, also supported by the similarity of the HPAI A(H5N1) viruses in those animals with those present locally in gulls. However, the finding of identical virus in several separate farms also indicates possible between-farm transmission. So far, based on whole genome sequencing results, within-farm virus transmission cannot be excluded. Preliminary serological results suggest that some animals in contact with cases may be affected without the presence of clinical signs of infection.
- Two new species, the northern fur seal in Russia and the southern elephant seal in Argentina, were found infected by HPAI viruses for the first time.
- In wild mammals, HPAI virus detections were reported in red fox (Finland, Latvia, Norway, Sweden, United Kingdom), seals (Argentina, Denmark, Germany, Russia, Uruguay), Eurasian otter (Finland) and South American sea lions (Argentina, Uruguay).
- Mass mortality events in South American sea lions and other marine mammal species continued to be reported from South America. The presence of HPAI A(H5N1) virus in South American sea lions and birds in Tierra del Fuego, at the southern tip of South America, poses a substantial risk of virus spread to wildlife in Antarctica.
- The ongoing and Europe-wide HPAI A(H5N1) epidemic in seabirds represents a risk for marine mammals along the European coastlines, including harbour seals that breed in the international Wadden Sea as well as wild and domestic carnivores sharing the same habitat. This is underlined by recent detections of HPAI A(H5N1) virus in harbour seals in Germany and Denmark.
- The HPAI A(H5N1) viruses detected in wild mammals in Europe during summer belonged to the genotypes BB and AB, the virus detected in cats in Poland to the CH genotype, and the virus detected in fur animal farms in Finland to the BB genotype.
- The high percentage (about 47%) of viruses collected from mammalian species containing molecular markers of mammalian adaptation in the PB2 protein indicates that these mutations can be rapidly acquired by the virus during infection in mammalian species. Viruses containing such mutations may have a greater zoonotic potential.
- To date, no key mutations associated with the switch in the virus binding preference from avian- to human-type receptors were identified in the A(H5) virus collected from mammalian species in Europe. However, matter of concern is the detection of a mutation that could change the receptor binding preference from avian to human in a A(H5N6) clade 2.3.4.4b virus that was collected in farmed dogs in China.

### 3.3 Humans

- Continued sporadic human avian influenza A(H3N8), A(H5N1), A(H5N6) and A(H9N2) virus detections or (severe) infections have been reported from different countries globally since the beginning of 2023.
- Severe and fatal infections in humans have so far been mainly related to unprotected exposure to sick and dead poultry, particularly in backyard settings, as well as to contaminated environment or to live bird markets.
- No symptomatic human cases have been detected in the EU despite the likely large number of exposure events over the last years.

## 4. Options for response

### 4.1 Birds

- Active surveillance in wild birds, especially in those that silently maintain HPAI viruses in the wild (e.g. waterfowl), is indicated to improve knowledge on HPAI viruses circulating in wild bird populations. Specific wild bird species that may be targeted for this purpose are, among others, ducks (e.g. dabbling ducks) and seabirds (e.g. gulls and terns).
- Species identification remains of utmost importance for the correct interpretation of passive surveillance efforts in wild birds. Species identification may be difficult in young birds but could be improved by genetic analyses on tissue sample or feathers, or with the collaboration of ornithologists to whom photos of the carcasses found may be sent.
- In consideration of the upcoming autumn migratory season, preparedness and prevention strategies continue to be priority measures to be implemented, primarily in high density poultry areas to avoid secondary spread. Protection of poultry from wild birds should be targeted. Also, despite lifting of the housing order in many countries, it may be prudent to keep poultry housed in the coming period in geographic regions where HPAI A(H5) virus is present in wild bird populations.
- Timely generation and sharing of genome sequence data from avian influenza viruses is of utmost importance to promptly detect the emergence of viruses with mutations associated with increased zoonotic potential, resistance toward antiviral drugs or different antigenic properties, whose biological characteristics should be further evaluated. Genetic data are also instrumental to track the virus spread and identify novel incursions of viruses which may represent a threat for human or animal health.

### 4.2 Mammals

- Increased surveillance of HPAI viruses in wild (e.g. red foxes) and free-roaming domestic carnivores (e.g. cats and dogs) continues to be recommended, for evaluating both the level of virus infections in these species and the risk of emergence and transmission of mammal-adapted viruses. Such surveillance efforts should prioritise domestic mammals present in or around HPAI-affected poultry establishments and those in possible contact with infected poultry, wild birds or other mammals. Research activities to investigate the role of asymptomatic mammals in maintaining HPAI viruses and driving their evolutionary dynamics, e.g.

by serological surveillance in farmed mammals (particularly American minks, foxes and common raccoon dogs), are recommended.

- Biosecurity should be improved in fur animal farms, where protection of farmed mammals from wild birds (especially seabirds and waterfowl) should be prioritised.
- Given the risk of mammalian adaptation of avian influenza viruses circulating in farmed mammals (e.g. fur animals), prompt culling of those animals present in HPAI virus-affected farms is indicated.
- Disease dynamics associated with HPAI virus infection during mass mortality events in mammal species should be thoroughly investigated. Testing a sufficient number of animals and assuring a prompt generation and sharing of viral sequences are of utmost importance to shed light on the virus origin, evolution and possible transmission between individuals. The latter is also indicated to timely assess the pandemic risk.
- More accurate and timely reporting of HPAI virus detections in mammals is recommended in a way that reliable numbers of infected animals could be used as quantitative information for risk assessment.
- It is recommended to avoid exposure of domestic cats and dogs, and in general carnivore pets, to dead or diseased animals (mammals and birds), and to avoid feeding cats, dogs and other domestic carnivores offal and raw meat coming from farms in areas in which circulation of HPAI virus is reported.

### 4.3 Humans

- People should avoid contact with sick or dead animals and inform authorities or veterinarians if they see dead birds or other animals to coordinate save removal and control measures.
- Appropriate personal protective equipment should be used when in contact with potentially infected animals.
- People exposed to sick or dead birds, infected mammals and contaminated environment should be followed-up for 10–14 days after last exposure and tested immediately following the onset of respiratory or any other symptoms to identify transmission events early. Following exposure to infected mammals, testing can also be considered without indications of onset of symptoms in the exposed people.
- During winter months, when seasonal influenza viruses are circulating in the population, testing and subtyping approaches for avian influenza virus need to be proportionate to the epidemiological situation and the capacities of reference laboratories. Therefore, in areas with ongoing avian influenza outbreaks in poultry and detections in wild birds and other animals, a risk-based targeted approach, focussing on outbreaks and severe respiratory or unexplained neurological disease, is proposed (https://www.ecdc.europa.eu/en/publications-data/avian-influenza-infections-surveillance-eu-eea).
- Options for response and additional information are available on ECDC webpages: Avian influenza

## Appendix A – Terms of Reference

### A.1. Background and Terms of Reference as provided by the requestor

Avian influenza is an infectious viral disease in birds, including domestic poultry. Infections with avian influenza viruses in poultry cause two main forms of that disease that are distinguished by their virulence. The low pathogenic (LPAI) form generally only causes mild symptoms, while the highly pathogenic (HPAI) form results in very high mortality rates in most poultry species. That disease may have a severe impact on the profitability of poultry farming.

Avian influenza is mainly found in birds, but under certain circumstances infections can also occur in humans even though the risk is generally very low.

More than a decade ago, it was discovered that virus acquired the capability to be carried by wild birds over long distances. This occurred for the HPAI of the subtype A(H5N1) from South East and Far East Asia to other parts of Asia, Europe and Africa as well as to North America. In the current epidemic the extent of the wild bird involvement in the epidemiology of the disease is exceptional.

Since late October 2016 up to early February 2017, highly pathogenic avian influenza (HPAI) of the subtype A(H5N8) has been detected in wild migratory birds or captive birds on the territory of 21 Member States, namely Austria, Belgium, Bulgaria, Croatia, Czechia, Denmark, Finland, France, Germany, Greece, Hungary, Ireland, Italy, the Netherlands, Poland, Portugal, Slovakia, Slovenia, Spain, Sweden and the United Kingdom. In 17 Member States the virus has spilled over to poultry holdings leading also to lateral spread between holdings in a few Member States, in particular in those with a high density of duck and geese holdings where the poultry cannot sufficiently be protected against contacts with wild birds. A second HPAI subtype A(H5N5) has been detected in wild birds and recently also in poultry holdings in Germany.

The number of infected migratory wild birds found dead and the geographic extent of these findings are posing an immense threat for virus introduction into poultry or captive birds holdings as demonstrated by the high number of outbreaks (~700 as of 08/02/2017).

In the event of an outbreak of avian influenza, there is a risk that the disease agent might spread to other holdings where poultry or other captive birds are kept. As a result, it may spread from one Member State to other Member States or to third countries through trade in live birds or their products.

There is knowledge, legislation, technical and financial tools in the EU to effectively deal with outbreaks of avian influenza in poultry and captive birds. However, the very wide virus spread by wild birds and the increased risk of direct or indirect virus introduction into poultry or captive bird holdings has led to the largest HPAI epidemic in the EU so far. This situation calls for a reflection and evaluation how preparedness, risk assessment, early detection and control measures could be improved.

The Commission and Member States are therefore in need of an epidemiological analysis based on the data collected from the disease affected Member States. The use of the EFSA Data Collection Framework is encouraged given it promotes the harmonisation of data collection. Any data that is available from neighbouring third countries should be used as well, if relevant.

Therefore, in the context of Article 31 of Regulation (EC) No. 178/2002[11], EFSA should provide the technical and scientific assistance to the Commission based on the following Terms of Reference (TOR):

1) Analyse the epidemiological data on highly pathogenic avian influenza (HPAI) and low pathogenic avian influenza (LPAI), where co-circulating or linked within the same epidemic, from HPAI disease affected Member States.

2) Analyse the temporal and spatial pattern of HPAI and LPAI as appropriate in poultry, captive birds and wild birds, as well the risk factors involved in the occurrence, spread and persistence of the HPAI virus in and at the interface of these avian populations.

3) Based on the findings from the points above, describe the effect of prevention and control measures.

4) Provide for regular quarterly reports updating on the avian influenza situation within the Union and worldwide, in particular with a view to describe the evolution of virus spread from certain regions towards the EU. In case of significant changes in the epidemiology of avian influenza, these reports could be needed more frequently. These reports should in particular closely follow the developments of zoonotic avian influenza viruses (such as HPAI A(H5N6) and LPAI A(H7N9)) in collaboration with the European Centre for Disease Prevention and Control (ECDC).

### A.2 Interpretation of the Terms of Reference

In reply to ToR 1 and ToR 2, this scientific report gives an overview of the HPAI and LPAI outbreaks in poultry, captive and wild birds detected in Europe from 24 June to 1 September 2023 and reported by Member States and neighbouring countries to the ADIS or WOAH. Member States where avian influenza outbreaks have occurred in poultry have submitted additional epidemiological data to EFSA, that have been used to analyse the characteristics of the affected poultry establishments.

It was not possible to collect data for a risk factor analysis on the occurrence and persistence of HPAI virus within the EU. Risk factor analysis requires not only case-related information, but also data on the susceptible population (e.g. location of establishments, population structure), which should be collected in a harmonised manner across the EU. Limitations in data collection, reporting and analysis were explained in the first avian influenza overview report (EFSA, ECDC and EURL, 2017).

If HPAI outbreaks in poultry are detected in the EU, a description of the applied prevention and control measures (ToR 3) is given in the overview document provided by representatives from the affected Member States and is provided in Annex A. Information was collected for outbreaks that occurred from 10 June to 1 September 2023. The main topics covered are increasing awareness, release and repeal of housing orders, strengthening biosecurity, preventive culling, implementation of a regional standstill, bans on hunting and derogations from restriction zone implementation after a risk assessment.

Monitoring of the avian influenza situation in other countries (ToR 4) is based on data reported to WOAH. The description focuses only on findings of avian influenza viruses occurring in countries that are considered to be of epidemiological interest for the EU/EEA and the UK or of public health relevance, specifically on HPAI A(H5N1), HPAI A(H5N2), HPAI A(H5N5), HPAI A(H5N6), HPAI A(H5N8), HPAI/LPAI A(H7N9) and LPAI A(H9N2). The background and epidemiology, detections, phenotypic and genetic characterisations are described based on information from confirmed human, poultry and wild bird cases that

[11] Regulation (EC) No 178/2002 of the European Parliament and of the Council of 28 January 2002 laying down the general principles and requirements of food law, establishing the European Food Safety Authority and laying down procedures in matters of food safety. OJ L 31, 1.2.2002, pp. 1–24.

occurred from 24 June to 1 September 2023. Possible actions for preparedness in the EU are discussed.

This report mainly describes information that has become available since the publication of the EFSA report for the period April to June 2023 (EFSA, ECDC and EURL, 2023) and that might affect the interpretation of risks related to avian influenza introduction and/or spread in Europe.

## Appendix B – Data and Methodologies

### B.1 Data on animals

#### B.1.1 Overview of avian influenza outbreaks in Europe (ToR 1 and ToR 2)

Data on the avian influenza outbreaks that occurred in Europe from 24 June to 1 September 2023 submitted by Member States to the ADIS were taken into account for this report. Data extraction was carried on 1 September 2023. WOAH-WAHIS was consulted to complement the information for European countries not reporting HPAI notifications to ADIS. In addition, HPAI-affected European countries were asked to provide more detailed epidemiological data directly to EFSA on the avian influenza outbreaks that occurred in poultry up to 18 August 2023. Wild bird species have been categorised according to Table A.2 in Annex A, and the common and scientific names of wild bird species described in this report in relation to Europe are reported in Table A.3 in Annex A. The public GISAID's EpiFlu™ Database was accessed to download newly released avian influenza virus sequences.

The annexes to this Scientific Report are available on the EFSA Knowledge Junction community on Zenodo at: https://doi.org/10.5281/zenodo.8382823

#### B.1.2 Overview of avian influenza outbreaks in other countries not reporting via ADIS (ToR 4)

Data from WOAH-WAHIS on HPAI A(H5N1), A(H5N2), A(H5N5), A(H5N6), A(H5N8) and LPAI A(H7N9) in domestic and wild birds were used to describe and map the geographic distribution of avian influenza virus detections in domestic and wild birds in Africa, the Americas, Asia and Europe based on the observation dates. Data were retrieved on 1 September 2023 and extracted by EFSA. They were used and reproduced with permission. WOAH bears no responsibility for the integrity or accuracy of the data contained herein, but not limited to, any deletion, manipulation, or reformatting of data that may have occurred beyond its control.

#### B.1.2 Genetic characterisation of avian influenza viruses: description of the nomenclature of the HPAI A(H5) viruses used in the document

The HA gene of clade 2.3.4.4 A(H5) viruses has rapidly evolved since the most recent official update of the nomenclature of the A/goose/Guangdong/1/1996-lineage H5Nx virus (Smith et al., 2015). This clade emerged in China in 2008 and since then it has acquired various neuraminidase subtypes, including N1, N2, N3, N4, N5, N6 and N8, by reassortments with other avian influenza viruses from different regions, and has evolved into several subgroups. While a revised nomenclature of clade 2.3.4.4 viruses is pending, in previous reports we used the genetic clustering described in 2018 by Lee and co-authors, who recognised four groups (a–d) within clade 2.3.4.4 (Lee et al., 2018). Recently, an update to the unified nomenclature for clade 2.3.4.4 A(H5) viruses has been proposed by the WHO and eight genetic groups (a–h) have been recognised. To align the nomenclature system between international organisations this classification has been adopted for this report. Based on this proposed clustering, A(H5) viruses of clades 2.3.4.4a and d–h have mainly been circulating in poultry in Asia, while clades 2.3.4.4b and 2.3.4.4c have spread globally through wild bird migrations during 2014–2015 (2.3.4.4c) and from 2016 to the present day (2.3.4.4b). A list with the distribution of the different genetic clades reported by countries globally from birds, humans and the environment has been published by WHO in February 2023 (WHO, 2023).

### B.2 Data on humans

The numbers of human cases caused by infection with avian influenza viruses were collected by ECDC. Multiple sources are scanned regularly as part of Epidemic Intelligence activities at ECDC to collect information on laboratory-confirmed human cases. Data were extracted and line lists developed to collect case-based information on virus type, date of disease onset, country of reporting, country of exposure, sex, age, exposure, clinical information (hospitalisation, severity) and outcome. All cases included in the line list and mentioned in the document have been laboratory-confirmed. Data are continuously checked for double entries and validity. The data on human cases cover the full period of time since the first human case was reported. Therefore, data on human cases refer to different time periods and are included irrespective of whether there have been any new human cases during the reporting period.
